# Essential Oils of *Laurus nobilis* L.: From Chemical Analysis to In Silico Investigation of Anti-Inflammatory Activity by Soluble Epoxide Hydrolase (sEH) Inhibition

**DOI:** 10.3390/foods13142282

**Published:** 2024-07-20

**Authors:** Francesca Fantasma, Vadym Samukha, Michela Aliberti, Ester Colarusso, Maria Giovanna Chini, Gabriella Saviano, Vincenzo De Felice, Gianluigi Lauro, Agostino Casapullo, Giuseppe Bifulco, Maria Iorizzi

**Affiliations:** 1 Department of Biosciences and Territory, University of Molise, Contrada Fonte Lappone, 86090 Pesche, IS, Italy; fantasma@unimol.it (F.F.); v.samukha@studenti.unimol.it (V.S.); saviano@unimol.it (G.S.); defelice@unimol.it (V.D.F.); iorizzi@unimol.it (M.I.); 2 Department of Pharmacy, University of Salerno, Via Giovanni Paolo II 132, 84084 Fisciano, SA, Italy; mialiberti@unisa.it (M.A.); ecolarusso@unisa.it (E.C.); glauro@unisa.it (G.L.); casapullo@unisa.it (A.C.)

**Keywords:** *Laurus nobilis* L., essential oil, anti-inflammatory effect, soluble epoxide hydrolase (sEH)

## Abstract

*Laurus nobilis* L. is commonly used in folk medicine in the form of infusion or decoction to treat gastrointestinal diseases and flatulence as a carminative, antiseptic, and anti-inflammatory agent. In this study, the essential oil (EO) composition of wild-grown *L. nobilis* L. leaves collected from seven different altitudinal locations in the Molise region and adjacent regions (Abruzzo and Campania) was investigated. EOs from the leaves were obtained by hydrodistillation and analyzed by GC-FID and GC/MS, and 78 compounds were identified. The major oil components were 1,8-cineol (43.52–31.31%), methyl-eugenol (14.96–4.07%), α-terpinyl acetate (13.00–8.51%), linalool (11.72–1.08%), sabinene (10.57–4.85%), α-pinene (7.41–3.61%), eugenol (4.12–1.97%), and terpinen-4-ol (2.33–1.25%). Chemometric techniques have been applied to compare the chemical composition. To shed light on the nutraceutical properties of the main hydrophobic secondary metabolites (≥1.0%) of laurel EOs, we assessed the in vitro antioxidant activities based on 2,2-diphenyl-1-picrylhydrazyl (DPPH•) radical scavenging activity and the reducing antioxidant power by using a ferric reducing power (FRAP) assay. Furthermore, we highlighted the anti-inflammatory effects of seven EOs able to interfere with the enzyme soluble epoxide hydrolase (sEH), a key enzyme in the arachidonic acid cascade, in concentrations ranging from 16.5 ± 4.3 to 8062.3 ± 580.9 mg/mL. Thanks to in silico studies, we investigated and rationalized the observed anti-inflammatory properties, ascribing the inhibitory activity toward the disclosed target to the most abundant volatile phytochemicals (≥1.0%) of seven EOs.

## 1. Introduction

Oxidative stress is a complex phenomenon in living organisms marked by an imbalance between the generation of free radicals (ROS) and the capacity to remove these reactive species from the body through endogenous and exogenous antioxidants. At low levels, ROS act as signal transduction molecules that stimulate cellular processes, while also offering cellular protection [1]. When ROS are present at high concentrations and are not adequately neutralized, they can damage cellular structures and biomolecules such as lipids, proteins, and DNA. This ultimately leads to the development of many illnesses [2,3]. Thus, researchers from all over the world are interested in finding natural antioxidants that can counteract the effects of free radicals in the context of global health. According to the World Health Organization (WHO), chronic diseases are the main threat to human health [3], and inflammation-related diseases are expected to increase over the next 30 years gradually [4]. Many studies indicate that several disorders, including cancer, heart disease, diabetes, arthritis, arteriosclerosis, Alzheimer’s disease, and immune system decline, are brought on by chronic inflammation [5]. The inflammatory cascade appears to be a crucial new target for the prevention of many disease conditions [6]. Many human pathologies can be traced back to the inflammatory mediators of the arachidonic acid cascade generated by lipoxygenases (LOXs), cyclooxygenases (COXs), and the cytochrome P450 pathway, in which soluble epoxide hydrolase (sEH) is involved [7]. sEH is a homodimeric enzyme found in numerous organs, including the brain, liver, and kidneys, that hydrolyzes epoxyeicosatrienoic acids (EETs), epoxyeicosatetraenoic acids (EEQs), and epoxydocosapentaenoic acids (EDPs) into their corresponding diols [8]. Emerging data indicate that blocking sEH leads to high levels of EETs, which possess anti-inflammatory properties and can hinder the onset of inflammation, hypertension, atherosclerosis, heart failure, fatty liver disease, and cancer [9,10,11]. Considering the remarkable advantages that can be obtained by blocking the activity of sEH, several synthetic ligands, characterized by urea and amide groups, have been prepared, which represent the most popular and potent class of sEH inhibitors [11,12].

Medicinal and aromatic plants offer an excellent opportunity for the identification of natural products to be used for pharmaceutical and nutraceutical purposes based on knowledge gained through local traditions [8,13], and sometimes, these products can ensure the health and well-being of the consumer by replacing synthetic drugs. Notably, a variety of potent sEH inhibitors have been isolated from natural sources, such as some natural urea-containing compounds isolated from *P. brazzeana* and *L. meyenii*, kaempferol and apigenine extracted from *T. hemsleyanum*, cimiciphenone and cimiracemate A obtained from *C. dahurica* and *Gentiana scabra*, and desoxyaloin isolated from *Aloe* [11].

Antioxidants can influence the immune response through several molecular mechanisms: by inducing the suppression of pro-inflammatory cytokines and inhibiting signaling pathways and key enzymes involved in immune processes [14,15,16]. In this context, many studies have shown that essential oils are endowed with pharmacological properties that make them useful in the management of diseases associated with oxidative stress [17].

*Laurus nobilis* L. has traditionally been used in folk medicine for its beneficial effects on health, which can now be scientifically explained by various biological activities identified in the extracts of its leaves, fruits, flowers, and roots, as well as in its essential oil [18,19,20,21,22].

*Laurus nobilis* L. (Lauraceae family), also known as bay laurel, true, daphne, Roman laurel, just laurel, or sweet bay, is an evergreen shrub or tree native to Europe and Mediterranean countries. Dried leaves and essential oils are the main commercial products on the market. Because of their flavor and fragrance, the leaves are used in cooking as a spice to improve the taste in red meat, fish, etc., and also in vegetarian dishes, according to traditional local recipes, not only in the cuisine of the Mediterranean area but also of many Asian countries [21,23,24,25,26].

In traditional medicine, the leaves and fruits have been used orally since ancient times for various gastrointestinal diseases, as well as for flatulence as a carminative, diaphoretic, antiseptic activity and against rheumatism, coughs, heart disease, diarrhoea [27,28,29]. Dried bay laurel and its infusions have been found to be a natural remedy for lowering blood sugar levels and protecting against fungal and bacterial infections [21] or for the treatment of various neurological, dermatological, and urological disorders [30,31]. In folk medicine, the essential oil from bay leaves is also recommended for the treatment of epilepsy, neuralgia, and parkinsonism [32,33].

The chemical composition of the leaves, fruits, flowers, and seeds of *L. nobilis* L. shows the presence of volatile components, sesquiterpenes, alkaloids, minerals, vitamins, sugars, polysaccharides, organic acids, tocopherols, and a wide range of polyphenols including different flavonoids, phenolic acids, tannins, and lignans [18,21,28].

Anti-inflammatory properties, with the inhibition of nitric oxide production, have been found in some megatigmane and sesquiterpene components isolated from leaf extracts [28,34,35], while several polyphenols, largely responsible for antioxidant activity, were highlighted in *L. nobilis* L. [18,20,36,37].

In the apolar extracts of *L. nobilis* L. leaves, sesquiterpene lactones such as costunolide and dehydrocostus lactone are the most abundant [38,39]. They exhibit anti-inflammatory activity, with the inhibition of NO production [34,40], and possess antibacterial and immunomodulatory effects [41]. In the last decade, the cytotoxic activity of these compounds against various cancer cell lines in vitro and in vivo has been actively studied [13,28,42,43,44,45,46,47].

Recently, two diglycosides isolated from laurel (laurusides) were investigated by molecular dynamics as potential ligands toward a well-preserved and crucial target, the 3C-like protease (M^pro^) of wild-type SARS-CoV-2 and Omicron variant in the context of new anti-β-coronavirus drug discovery [48].

The commercial value of *L. nobilis* L. derives from its essential oil, and its applications extend from food to cosmetics and drugs [49]. Essential leaf oil is composed of an intricate blend of constituents, some of them in larger amounts or with varying proportions, each contributing to a distinctive scent. The great number of phytochemicals in the EOs exerts physiological effects and therapeutic potential, including anticonvulsant [33], in vitro antibacterial [50], antifungal [51], antidiabetic [41,52,53], analgesic and anti-inflammatory [32], anticancer [54,55], neuroprotective [56], anticholinergic [57], and antioxidant effects [49,58,59], acaricidal [60] activities, and insecticidal effects [61]. Allergic contact dermatitis with laurel essential oil has been observed in rare cases [62]. Recently, the use of EOs has been proposed for applications in the food industry [25].

Essential oils (EOs) are gradually becoming more popular natural ingredients in the cosmetics industry due to their olfactory characteristics and the numerous beneficial properties of their components [17], including anti-inflammatory, antimicrobial, and antioxidant properties; so, they are recommended in the preparation of moisturizers, lotions, and cleansers in skin care cosmetics, etc. [63].

This study aims to investigate the EO composition of *L. nobilis* L. leaves grown in the wild in seven distinct altitudinal locations and their therapeutic potential. Specifically, five samples were gathered from the Molise area (LNMO) (central-southern Italy), and two samples were obtained from the surrounding regions of Abruzzo (LNAB) and Campania (LNCA).

Because of the complexity of volatile EO components, chemometric techniques have been applied, which can serve as a valuable alternative for characterizing the properties of individual constituents. These techniques enable the evaluation of multiple variables and their interactions and also allow for obtaining a single and comprehensive response for multiple dependent variables [64].

In light of the many applications in ethnomedicine and the important pharmacological activities found in EOs linked to their anti-inflammatory activity, this work aims (a) to perform the extraction and characterization of the chemical constituents of volatile EOs by GC-FID and GC-MS; (b) to compare the chemical composition using chemometric analysis; (c) to evaluate the in vitro antioxidant potential of EOs based on 2,2-diphenyl-1-picrylhydrazyl (DPPH•) radical scavenging activity and the reducing antioxidant power by using a ferric reducing power (FRAP) assay; (d) to investigate the soluble epoxide hydrolase (sEH) inhibitory activities of EOs using a fluorescent assay; and (e) to rationalize the inhibitory effect of the most abundant volatile phytochemicals (≥1.0%) of EOs on the crystal structure of sEH through in silico studies.

Thus, these results point toward the use of EOs for the treatment of inflammatory status, and interestingly, they are in line with a natural product-based fragment virtual screening. In fact, the most abundant metabolites of EOs (≥1.0%) could be promising candidates for the development of semi-synthetic sEH inhibitors endowed with unprecedented chemical scaffolds.

## 2. Materials and Methods

### 2.1. Plant Material

Fresh leaves of *L. nobilis* (LN), grown at different altitudes, were collected in April 2023, in the Molise area (central-southern Italy) at Petacciato (CB), Vivaio Forestale Regionale “Le Marinelle”, about 25 m above sea level (a.s.l.) (200 m away from the Adriatic Sea) (LNMO1), Campobasso (CB), 700 m a.s.l. (LNMO2), Isernia (IS), 420 m a.s.l. (LNMO3), Carpinone (IS), 630 m a.s.l. (LNMO4), Capracotta (IS), 1420 m a.s.l. (LNMO5), and in the neighboring regions of Abruzzo at Rosciano (Pescara), 150 m a.s.l. and a distance of 20 km from the Adriatic Sea (LNAB), and Campania at Santa Maria Capua Vetere (Caserta), about 35 m a.s.l. (LNCA).

Representative homogeneous samples of the population were collected during the balsamic time. The plants were identified by Prof. P. Fortini and a voucher specimen of each species was deposited at the Herbarium of University of Molise (Pesche, Isernia) under the registry numbers LNMO1-Petacciato-04-2023; LNMO2-CB-04-2023; LNMO3-IS-04-2023; LNMO4-Carpinone-04-2023; LNMO5-Capracotta-04-2023; LNAB-Rosciano-04-2023; LNCA-SMCVetere-04-2023.

### 2.2. Essential Oil Isolation

A defined quantity of leaves (800 g) of the seven collected *L. nobilis* samples was selected and cleaned by hand; the leaves were next separately subjected to hydrodistillation using Clevenger apparatus (i.e., Albrigi Inherba, Italy) for 3 h according to the standard procedure described in the Council of Europe [65,66].

The obtained essential oils were dried over anhydrous sodium sulfate to remove traces of water and then stored in dark vials at 4 °C prior to gas chromatography–mass spectrometry (GC-MS) analysis.

### 2.3. GC-FID Analysis and GC/MS Analysis

The characterization of the EO samples was performed using a gas chromatography system, followed by GC/MS analysis. The GC-FID analysis was carried out using a GC 86.10 Expander (Dani), equipped with an FID detector, an Rtx^®^-5 Restek capillary column (30 m × 0.25 mm i.d., 0.25 um film thickness) (diphenyl-dimethyl polysiloxane), a spilt/splitless injector heated to 250 °C, and a flame ionization detector (FID) heated to 280 °C. The column temperature was maintained at 40 °C for 5 min, then programmed to increase to 250 °C at a rate of 3 °C/min, and held, using an isothermal process, for 10 min. The carrier gas was He (2.0 mL/min); 1 μL of each sample was dissolved in n-hexane (1:500 n-hexane solution) and injected. All experiments were repeated three times.

The GC-MS analyses were performed on a Trace GC Ultra (Thermo Fisher Scientific, Waltham, MA, USA) gas chromatography instrument equipped with the same Rtx^®^-5 Restek capillary column (30 m × 0.25 mm i.d., 0.25 um film thickness) and coupled with an ion-trap (IT) mass spectrometry (MS) detector Polaris Q (Thermo Fisher Scientific, Waltham, MA, USA). A Programmed Temperature Vaporizer (PTV) injector and a PC with a chromatography station Xcalibur (Thermo Fisher Scientific, Waltham, MA, USA) were used. The ionization voltage was 70 eV; the source temperature was 250 °C; and full scan acquisition under positive chemical ionization was from *m*/*z* 40 up to 400 a.m.u. at 0.43 scan s-1. The GC conditions for the gas chromatography (GC-FID) analysis were the same as those described above.

### 2.4. Identification and Relative Percentage of EO Components

The identification of the EO components of all samples analyzed was based on the comparison of their Kovats retention indices (Exp RI) with the Kovats retention indices reported in the literature (Ref RI) [67]; the Kovats indices (Exp RI) were determined in relation to the retention time (t_R_) values of a homologous series of n-alkanes (C8–C20) injected in the same operating conditions as the samples under analysis, as reported in the literature [68,69].

Additionally, the MS fragmentation pattern of each single compound was compared with that from the NIST 02, Adams, and Wiley 275 mass spectral libraries [70,71]. The quantitative relative contents (%) of the sample components were computed as the average of the GC peak areas obtained in triplicate without any corrections [72]. For the quantitative purpose, all analytical standard components utilized (n-alkane C8–C20, α-pinene, β-pinene, 1,8-cineole, linalool, borneol, terpinen-4-ol, sabinene) were bought from Sigma Aldrich, St. Louis, MO, USA. The list of components is shown in Table 1.

### 2.5. Multivariate Analysis

The multivariate analysis was performed by Partial Least Square–Discriminant Analysis (PLS-DA), a “supervised” version of Principal Component Analysis (PCA), which is made aware of the class labels in its input [73]. The essential oils of all of the samples were analyzed in triplicate by GC-MS, and the integrated areas (% area) of the peaks of the metabolites detected were obtained. A data set with 21 observations (7 varieties of *Laurus nobilis*) and 78 variables (number of metabolites detected) was imported to MetaboAnalyst 6.0, selecting the statistical analysis (one factor). All of the variables were Pareto-scaled to reduce the relative importance of metabolites with high intensities by decreasing large fold changes more than small ones. Additionally, the impact of MS noise is reduced by Pareto scaling [74]. Moreover, the Variable Importance in Projection (VIP) analysis by MetaboAnalyst showed the most important metabolites that contribute to the separation of the clusters, considering metabolites with a VIP value > 1.

### 2.6. Determination of Total Phenolics from Samples

The total phenolic content was determined according to Folin–Ciocalteu’s methods with slight modifications [75]. Briefly, to 0.100 mL of diluted essential oil (0.01 mL EO in 10.0 mL methanol), we added 0.400 mL of deionized water and 1.0 mL of Folin–Ciocalteu reagent (1:10) (Titolchimica, Rovigo, Italy). After 10 min, 2.0 mL of Na_2_CO_3_ (7.5%) (Acros, Geel, Belgium) was added to obtain a final volume of 3.5 mL. The solutions were placed in a dark environment and kept at room temperature for 60 min. The solutions were then subjected to centrifugation at a speed of 4000 revolutions per minute for 3 min. The resulting liquid above the sediment was collected and placed into cuvettes for measurement of absorbance using a VIS spectrophotometer (Shimadzu UV-1601 spectrophotometer, Shimadzu, Kyoto, Japan) at a wavelength of 765 nm. The total phenolic contents were calculated as the gallic acid equivalent (GAE) from a calibration curve of gallic acid standard solutions (range 1.0–10 μg/mL) and expressed as mg GAE (gallic acid equivalent) per g of EO sample. All of the experiments were carried out in triplicate.

### 2.7. Antioxidant Capacity Assays

#### 2.7.1. DPPH Radical Scavenging Activity

The ability of the essential oil to scavenge the DPPH radical was tested as previously described [76], with some modifications (essential oil diluted in absolute ethanol). In particular, 1 mL of essential oil at different concentrations diluted in ethanol (as reported in Table 2) was added to 1 mL of a freshly prepared DPPH• ethanolic solution (27 μg/mL dil.). The reaction mixture was incubated in the absence of light for 30 min. During this time, the DPPH radical undergoes reduction upon interaction with an antioxidant molecule, resulting in a noticeable change in color. The alterations in color were measured as absorbance at a wavelength of 517 nm using a Shimadzu UV-1601 spectrophotometer (Shimadzu, Kyoto, Japan), with ethanol serving as a reference. The mixture of 1 mL of the DPPH solution and 1 mL of ethanol was taken as the control product.

As an indication, the ascorbic acid (range 2.0–600 μg/mL) as a standard, known for its anti-radical effect, was tested in parallel (positive control). The antioxidant activity was quantified using IC_50_, which is the concentration (mg/mL) of the extract needed to neutralize 50% of DPPH free radicals. The value was determined by linear regression analysis of the dose–response curve, which was generated by plotting the radical scavenging activity against the concentration of the extract within the range of 0.1–1.5 mg/mL.

In order to estimate the IC_50_, various levels of inhibition were calculated as follows:

% Radical Scavenging Activity = [1 − (A_sample_/A_control_)] × 100

where A_control_ is the absorption of 1 mL of DPPH• and 1 mL of ethanol, and A_sample_ is the absorption of the sample. As for the inhibitory concentrations (IC_50_), they are calculated from the curves of linear regression. Tests were carried out in triplicate, and the results were expressed as the mean of the obtained IC_50_ values ± standard error (SE).

#### 2.7.2. Ferric Reducing Power (FRAP)

The reductive activity of the iron of our essential oil was determined according to the method described by Benzie et al. [77], with modifications. The active FRAP reagent was created by combining acetate buffer (300 mM, pH 3.6) with TPTZ (2,4,6-tripyridyl-s triazine) solution (10 mM in 40 mM HCl) and FeCl_3_ × 6 H_2_O (20 mM) at a ratio of 10:1:1. A The total extract (200 μL) was reacted with 1.8 mL of FRAP and incubated at 37 °C for 30 min. The absorbance was measured at 593 nm (Shimadzu UV-1601 spectrophotometer, Shimadzu, Kyoto, Japan). As an indication, ascorbic acid (1.2 μg/mL) was tested in parallel. However, an increase in absorbance corresponds to an increase in the reducing power of the extracts tested. The measurement of antioxidant activity in vitro was conducted by employing a standard Trolox curve within a concentration range from 4 to 25 μM. The results were quantified in terms of millimoles of Trolox equivalents per gram of EO sample.

### 2.8. Cell-Free sEH Activity Assay

Each of the seven EOs samples was tested in single-dose triplicate mode at a final concentration of 10 μg/μL against sEH using the Soluble Epoxide Hydrolase Inhibitor Screening Assay Kit (10011671), and then, the IC_50_ values were calculated for each compound. sEH protein was diluted in bis-Tris buffer (25 mM, pH 7) and pre-incubated with the test samples or the reference compound (AUDA, 12-(3-adamantan-1-yl-ureido) dodecanoic acid) or vehicle (2.5% EtOH) for 15 min at 25 °C. The reaction was started by the addition of 5 μL of substrate (PHOME, 10 μM) to obtain a final concentration of 0.25 μM. When the epoxide moiety of PHOME undergoes hydrolysis by epoxide hydrolase, intramolecular cyclization takes place, leading to the release of a cyanohydrin under basic conditions. The cyanohydrin rapidly decomposes into cyanide ions and the highly fluorescent 6-methoxy-2-naphthaldehyde, which can be analyzed. The signal was detected (λ_ex_ 330 nm, λ_em_ 465 nm) on an EnSpire™ Multimode Plate Reader (PerkinElmer, California, USA).

### 2.9. In Silico Studies

The X-ray crystal structure of sEH in complex with its ligand, 2-({[2-(adamantan-1-yl)ethyl]amino}methyl)phenol, was downloaded from RCSB PDB (ID: 4Y2X [78]) and prepared using Protein Preparation Wizard [79,80] (Schrödinger Suite); the solvent and co-complexed compound were removed, cap termini were included, all hydrogen atoms were added, and bond orders were assigned. The grid box for molecular docking experiments was accounted for using a co-crystallized ligand as a guide to define the centroid of the active site. The final coordinates of the grid center were −17.54 (x), −9.70 (y), and 66.48 (z), and the inner and outer box dimensions were 10 × 10 × 10 and 24.58 × 24.58 × 24.58, respectively.

The 2D structures of the main chemical constituents of EOs were downloaded from PubChem in .sdf format and prepared using LigPrep software (version 5.7, Schrödinger, LLC, New York, NY, USA, 2021) [81,82,83,84,85], which allows for the generation of all of the possible tautomers and protonation states at pH = 7.4 (±0.1) and for the minimization of the structures obtained using the OPLS 2005 force field.

Glide software (version 9.0, Schrödinger, LLC, New York, NY, USA, 2021) [82,83,84,85,86] (Schrödinger Suite) was used for molecular docking experiments, which were conducted in the Extra-Precision (XP) mode. Specifically, during the initial docking phase, 10,000 poses were kept, and 800 conformations were selected for the minimization step with an energy threshold of 0.15 kcal/mol. Finally, a maximum of 20 poses were retained for analysis to examine the binding mode.

## 3. Results and Discussion

### 3.1. Chemical Profile of Essential Oils

Many factors can influence the chemical composition of essential oils: geographical origin and harvest season, environmental conditions, the phenological growth stage of the plant, drying methods, extraction, and analysis conditions [25,87,88]. Therefore, each essential oil has its own unique chemical profile, and its biological activities are influenced by the co-presence of compounds that act in a synergetic or antagonistic manner [89].

The chemical compositions of the bay laurel EOs are provided in Table 1. The EOs qualitatively and quantitatively showed a high degree of metabolic variability.

The relative content analysis revealed that the predominant component found in all samples was 1,8-cineol (eucalyptol), with a content ranging from 43.52% to 31.31%, followed by methyl-eugenol (14.96–4.07%), α-terpinyl acetate (13.00–8.51%), linalool (11.72–1.08%), and sabinene (10.57–4.85%) as primary components.

**Figure 1:**
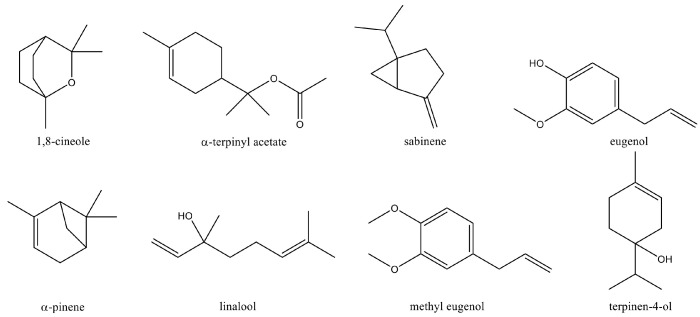
Major chemical components (≥1.0%) identified in EOs of *L. nobilis* L. leaves.

For contents ranging from 7.50% to 1.40%, we identified α-pinene (7.41–3.61%) and eugenol (4.12–1.97%), while β-pinene, α-terpineol, and terpinen-4-ol accounted for (3.81–1.38%), (2.91–0.91%), and (2.33–1.25%), respectively. Seventy-eight components have been identified, represented by bi- and tricyclic oxygenated monoterpenes (BMOs, from 45.38% to 32.38%), followed by bi- and tricyclic monoterpenes (BMs, from 23.66% to 15.79%) and other components such as phenylpropanoids (eugenol, methyl eugenol, elemicin, etc.) from 21.57% to 7.74%. In lower concentrations, monocyclic oxygenated monoterpenes (MMOs, from 17.73% to 12.9 8%) were identified (see Supplementary Materials Table S1). Differences in the composition of the EOs of wild-grown *L. nobilis* L. in other geographical areas have been reported. However, the main components identified in our samples are in line with the metabolites found in EOs in Italy or in the Mediterranean basin, including a high level of 1,8-cineole [27,90,91]. Figure 1 shows the major constituents of *L. nobilis* EOs.

**Table 1:** A list of volatile phytochemicals of the essential oils (EOs) isolated from the leaves of seven varieties of *Laurus nobilis* L.

N.	t_R (min)_	Exp RI	Ref RI	Class	Compound	Area (%) ± SD						
						LNMO1	LNMO2	LNMO3	LNMO4	LNMO5	LNCA	LNAB
1	11.17	931	930	BM	*α*-Thujene	0.46 ± 0.06	0.35 ± 0.01	0.42 ± 0.08	0.33 ± 0.09	0.42 ± 0.05	0.47 ± 0.03	0.42 ± 0.04
2	11.48	937	939	BM	*α*-Pinene	4.83 ± 0.09	5.17 ± 0.1	7.41 ± 0.25	3.61 ± 0.21	4.80 ± 0.07	4.73 ± 0.04	4.06 ± 0.09
3	12.19	951	954	BM	Camphene	0.50 ± 0.07	0.90 ± 0.1	0.70 ± 0.06	0.11 ± 0.02	0.10 ± 0.01	0.10 ± 0.02	0.17 ± 0.03
4	13.62	976	975	BM	Sabinene	6.70 ± 0.38	9.84 ± 0.14	10.57 ± 0.38	9.12 ± 0.56	4.85 ± 0.28	10.2 ± 0.34	7.56 ± 0.11
5	13.71	979	979	BM	*β*-Pinene	3.77 ± 0.28	2.96 ± 0.33	3.81 ± 0.39	2.44 ± 0.35	2.47 ± 0.11	3.00 ± 0.24	2.61 ± 0.15
6	14.6	992	990	AM	Myrcene	0.38 ± 0.03	0.88 ± 0.01	0.74 ± 0.08	1.02 ± 0.06	0.56 ± 0.06	1.01 ± 0.05	0.7 ± 0.06
7	15.15	1003	1002	MM	*α*-Phellandrene	0.79 ± 0.06	0.04 ± 0.02	0.06 ± 0.01	0.03 ± 0.02	0.08 ± 0.01	0.16 ± 0.01	0.12 ± 0.01
8	15.48	1010	1011	BM	*δ*-3-Carene	0.34 ± 0.05	0.10 ± 0.01	0.58 ± 0.02	-	0.44 ± 0.02	0.47 ± 0.03	0.34 ± 0.07
9	15.81	1016	1017	MM	*α*-Terpinene	0.45 ± 0.04	0.41 ± 0.02	0.37 ± 0.02	0.41 ± 0.02	0.37 ± 0.02	0.47 ± 0.04	0.46 ± 0.06
10	16.36	1029	1024	MM	*o*-Cymene	0.63 ± 0.07	-	-	-	-	-	-
11	16.52	1035	1031	BMO	1,8-Cineole	41.01 ± 0.3	43.52 ± 0.35	39.9 ± 1.17	40.7 ± 0.23	31.31 ± 0.17	42.39 ± 0.83	33.72 ± 0.14
12	17.57	1052	1050	AM	(*E*)-*β*-Ocimene	0.05 ± 0.01	0.13 ± 0.01	0.15 ± 0.01	0.27 ± 0.02	0.11 ± 0.01	0.10 ± 0.01	0.11 ± 0.01
13	18.03	1061	1059	MM	*γ*-Terpinene	0.79 ± 0.03	0.74 ± 0.01	0.75 ± 0.04	0.68 ± 0.03	0.71 ± 0.01	0.86 ± 0.01	0.83 ± 0.02
14	18.48	1070	1070	BMO	*cis*-Sabinene hydrate	0.22 ± 0.01	0.33 ± 0.02	0.32 ± 0.01	0.38 ± 0.01	0.22 ± 0.01	0.26 ± 0.01	0.21 ± 0.01
15	19.53	1088	1088	MM	Terpinolene	0.37 ± 0.03	0.28 ± 0.02	0.3 ± 0.02	0.30 ± 0.03	0.25 ± 0.02	0.44 ± 0.01	0.32 ± 0.02
16	20.08	1098	1098	BM	*trans*-Sabinene hydrate	0.18 ± 0.02	0.20 ± 0.03	0.17 ± 0.01	0.18 ± 0.03	0.06 ± 0.01	0.13 ± 0.01	0.11 ± 0.01
17	20.3	1102	1096	AMO	Linalool	1.08 ± 0.05	3.93 ± 0.06	1.09 ± 0.02	2.80 ± 0.03	11.72 ± 0.13	3.44 ± 1.85	10.29 ± 0.14
18	21.24	1123	1120	BMO	Sabina ketone dehydro	0.08 ± 0.01	0.09 ± 0.01	0.06 ± 0.01	0.08 ± 0.01	0.07 ± 0.01	0.07 ± 0.01	0.08 ± 0.01
19	21.51	1129	1126	MMO	α-Campholenal	0.01 ± 0.01	-	-	-	-	-	-
20	21.65	1132	1132	AM	(*allo*)-Ocimene	0.01 ± 0.01	0.01 ± 0.01	0.02 ± 0.01	0.01 ± 0.01	0.01 ± 0.01	0.01 ± 0.01	-
21	22.07	1141	1139	MBO	*trans*-Pinocarveol	0.04 ± 0.01	-	-	-	-	-	-
22	22.17	1143	1144	AM	(*neo-allo*)-Ocimene	0.08 ± 0.01	0.03 ± 0.02	0.03 ± 0.01	0.04 ± 0.01	0.06 ± 0.01	0.04 ± 0.01	0.04 ± 0.01
23	23.83	1156	1154	OT	Isobutyl hexanoate	-	0.01 ± 0.01	-	0.02 ± 0.01	0.08 ± 0.01	-	-
24	24.04	1160	1164	AMO	*(Z*)-Isocitral	0.04 ± 0.01	-	-	-	-	-	-
25	23.44	1168	1169	BMO	Borneol	0.19 ± 0.01	0.37 ± 0.02	0.17 ± 0.01	0.02 ± 0.01	0.04 ± 0.01	0.03 ± 0.01	0.05 ± 0.01
26	23,52	1170	1171	MMO	(*neoiso*)-Isopulegol	0.22 ± 0.02	0.20 ± 0.02	0.26 ± 0.01	0.28 ± 0.01	0.14 ± 0.01	0.15 ± 0.01	0.2 ± 0.01
27	24.01	1179	1177	MMO	Terpinen-4-ol	2.33 ± 0.11	1.45 ± 0.03	1.46 ± 0.01	1.25 ± 0.01	1.74 ± 0.01	1.58 ± 0.05	1.69 ± 0.01
28	24.74	1192	1188	MMO	*α*-Terpineol	2.27 ± 0.10	1.00 ± 0.05	2.55 ± 0.04	2.91 ± 0.01	0.91 ± 0.01	1.31 ± 0.05	1.92 ± 0.03
29	25.05	1198	1196	MMO	Methyl chavicol	0.14 ± 0.01	0.07 ± 0.01	0.10 ± 0.01	0.17 ± 0.01	0.16 ± 0.01	0.93 ± 0.01	0.11 ± 0.01
30	26.52	1232	1229	AMO	Nerol	0.22 ± 0.01	0.20 ± 0.01	0.23 ± 0.01	0.10 ± 0.01	0.19 ± 0.01	0.10 ± 0.01	0.15 ± 0.01
31	27.81	1260	1257	AMO	Linalool acetate	0.06 ± 0.02	0.16 ± 0.04	0.05 ± 0.01	0.05 ± 0.01	0.73 ± 0.04	0.22 ± 0.01	0.57 ± 0.01
32	27.88	1261	1262	OT	(*Z*)-Cinnamyl alcohol	0.10 ± 0.02	0.04 ± 0.01	-	0.01 ± 0.01	0.16 ± 0.03	-	-
33	28.48	1274	1270	OT	(*E*)-Cinnamaldehyde	0.02 ± 0.01	0.02 ± 0.01	0.03 ± 0.01	0.03 ± 0.01	0.01 ± 0.01	-	0.02 ± 0.01
34	28.7	1278	1282	BMO	(*cis*)-Verbenyl acetate	0.14 ± 0.01	0.11 ± 0.05	0.03 ± 0.01	0.06 ± 0.01	0.23 ± 0.01	0.13 ± 0.01	0.12 ± 0.01
35	29.16	1288	1288	BMO	Bornyl acetate	0.59 ± 0.01	0.82 ± 0.15	0.25 ± 0.36	0.16 ± 0.02	0.13 ± 0.01	0.13 ± 0.01	0.19 ± 0.01
36	29.5	1294	1290	BMO	(*trans*)-Sabinyl acetate	0.16 ± 0.01	0.14 ± 0.01	0.02 ± 0.01	0.03 ± 0.01	0.28 ± 0.01	0.05 ± 0.01	0.04 ± 0.01
37	29.63	1297	1299	MMO	Carvacrol	0.02 ± 0.01	-	-	-	-	-	-
38	30.58	1319	1317	MMO	*δ*-Terpinyl acetate	0.60 ± 0.02	0.46 ± 0.11	0.44 ± 0.01	0.42 ± 0.01	0.48 ± 0.01	0.76 ± 0.01	0.4 ± 0.01
39	31.39	1338	1338	MS	*δ*-Elemene	0.05 ± 0.01	0.06 ± 0.01	0.21 ± 0.05	0.10 ± 0.03	0.08 ± 0.01	0.21 ± 0.01	0.27 ± 0.12
40	32.12	1355	1349	MMO	*α*-Terpinyl acetate	10.2 ± 0.24	11.43 ± 0.21	8.51 ± 1.31	9.16 ± 0.04	9.55 ± 0.03	13.00 ± 0.34	9.31 ± 0.12
41	32.45	1363	1359	MMO	Eugenol	4.12 ± 0.08	3.30 ± 0.11	3.41 ± 0.15	1.97 ± 0.12	4.05 ± 0.01	2.65 ± 0.02	2.81 ± 0.06
42	32.72	1368	1361	AMO	Neryl acetate	0.05 ± 0.01	0.06 ± 0.01	-	0.24 ± 0.01	0.10 ± 0.01	0.17 ± 0.01	0.09 ± 0.01
43	32.96	1374	1368	OT	Hydrocinnamyl acetate	0.04 ± 0.01	-	-	0.40 ± 0.01	-	-	-
44	33.1	1377	1376	BS	*a*-Copaene	0.01 ± 0.01	-	-	-	-	-	0.08 ± 0.01
45	33.73	1391	1390	BS	*iso*-Longifolene	0.02 ± 0.02	-	0.03 ± 0.01	-	-	0.08 ± 0.01	0.14 ± 0.02
46	33.83	1393	1390	MS	*β*-Elemene	0.32 ± 0.02	0.20 ± 0.03	0.34 ± 0.07	0.32 ± 0.08	0.29 ± 0.01	0.58 ± 0.01	0.79 ± 0.02
47	34.14	1399	1400	OT	Tetradecane	0.02 ± 0.01	0.13 ± 0.01	0.15 ± 0.02	0.10 ± 0.01	0.05 ± 0.01	0.13 ± 0.01	0.14 ± 0.01
48	34.57	1410	1403	MMO	Methyl eugenol	10.54 ± 0.31	4.35 ± 0.07	6.08 ± 0.14	14.5 ± 0.22	14.96 ± 0.05	4.07 ± 0.10	10.86 ± 0.06
49	34.97	1420	1419	BS	(*E*)-Caryophyllene	0.42 ± 0.14	0.34 ± 0.01	0.79 ± 0.02	0.75 ± 0.02	0.32 ± 0.01	0.57 ± 0.02	0.77 ± 0.01
50	35.79	1441	1441	BS	Aromadendrene	0.05 ± 0.01	0.01 ± 0.01	-	-	0.07 ± 0.01	-	0.19 ± 0.01
51	35.98	1445	1444	BS	*6,9*-Guaiadiene	0.03 ± 0.01	-	-	-	-	-	-
52	36.18	1449	1446	MMO	(*E*)-Cinnamyl acetate	-	0.03 ± 0.01	-	0.96 ± 0.02	0.09 ± 0.01	-	-
53	36.2	1451	1451	BS	*α*-Himachalene	0.03 ± 0.01	-	-	-	-	-	-
54	36.28	1453	1449	BS	Spirolepechinene	-	-	-	-	0.06 ± 0.01	-	-
55	36.39	1455	1454	MS	*α*-Humulene	0.05 ± 0.01	0.24 ± 0.01	0.12 ± 0.01	0.09 ± 0.01	0.08 ± 0.01	0.09 ± 0.01	0.13 ± 0.01
56	36.69	1462	1460	BS	*allo*-Aromadendrene	0.02 ± 0.01	0.01 ± 0.01	-	-	0.07 ± 0.01	-	0.09 ± 0.01
57	36.78	1463	1463	BS	*1(6),4-diene, cis*-Cadina	0.03 ± 0.01	-	-	-	-	-	-
58	37.37	1479	1479	BS	*γ*-Muurolene	-	-	-	-	0.06 ± 0.01	-	-
59	37.54	1482	1485	MS	Germacrene *D*	0.09 ± 0.01	0.03 ± 0.01	0.23 ± 0.03	0.10 ± 0.01	0.03 ± 0.01	0.30 ± 0.01	0.13 ± 0.01
60	37.74	1487	1490	BS	*β*-Selinene	0.08 ± 0.02	0.04 ± 0.01	0.05 ± 0.02	0.10 ± 0.01	0.20 ± 0.01	0.07 ± 0.01	0.34 ± 0.01
61	38.12	1496	1494	BSO	*epi*-Cubebol	0.10 ± 0.02	0.10 ± 0.01	0.22 ± 0.01	-	0.14 ± 0.01	-	0.09 ± 0.01
62	38.18	1497	1495	BS	*γ*-Amorphene	0.08 ± 0.02	0.15 ± 0.01	0.38 ± 0.08	0.27 ± 0.05	0.46 ± 0.04	0.26 ± 0.02	0.73 ± 0.03
63	38.62	1508	1512	BS	*δ*-Amorphene	0.04 ± 0.01	0.07 ± 0.01	0.12 ± 0.02	0.27 ± 0.07	0.07 ± 0.02	0.18 ± 0.01	0.18 ± 0.03
64	38.82	1515	1513	BS	*γ*-Cadinene	0.10 ± 0.01	0.08 ± 0.02	0.38 ± 0.01	-	0.12 ± 0.01	0.27 ± 0.01	0.18 ± 0.01
65	39.27	1525	1523	BS	*δ*-Cadinene	0.15 ± 0.02	0.13 ± 0.01	0.25 ± 0.02	0.11 ± 0.01	0.18 ± 0.01	0.46 ± 0.01	0.55 ± 0.02
66	39.57	1533	1534	BS	*trans*-Cadina-1,4-diene	0.06 ± 0.01	0.07 ± 0.01	0.15 ± 0.01	-	0.01 ± 0.01	-	0.05 ± 0.01
67	40.03	1545	1545	BS	*α*-Calacorene	0.05 ± 0.01	-	-	-	-	-	-
68	40.32	1552	1549	MSO	Elemol	0.10 ± 0.01	-	-	-	0.06 ± 0.01	-	-
69	40.72	1563	1557	MMO (OT)	Elemicin	1.19 ± 0.06	0.57 ± 0.03	0.63 ± 0.01	0.60 ± 0.03	2.05 ± 0.02	0.69 ± 0.01	1.19 ± 0.02
70	41.41	1580	1578	BSO	Spathulenol	0.44 ± 0.06	0.86 ± 0.16	1.76 ± 0.12	0.26 ± 0.02	0.94 ± 0.01	0.49 ± 0.03	0.78 ± 0.02
71	41.63	1585	1583	BSO	Caryophyllene oxide	0.41 ± 0.05	0.63 ± 0.12	0.83 ± 0.04	0.16 ± 0.01	0.33 ± 0.01	0.21 ± 0.01	0.36 ± 0.02
72	41.97	1594	1592	BSO	Viridiflor	0.04 ± 0.01	-	-	-	0.09 ± 0.01	-	-
73	42.21	1600	1600	OT	Hexadecane	0.08 ± 0.01	0.20 ± 0.06	0.28 ± 0.02	0.21 ± 0.01	0.12 ± 0.01	0.29 ± 0.04	0.33 ± 0.01
74	43.01	1622	1623	BSO	*10-epi-γ* Eudesmol	0.05 ± 0.02	-	-	-	0.07 ± 0.01	-	-
75	43.41	1633	1632	BSO	*γ*-Eudesmol	0.27 ± 0.01	-	-	-	-	-	-
76	44.17	1653	1653	BSO	*α*-Eudesmol	0.23 ± 0.01	0.08 ± 0.03	0.28 ± 0.02	0.11 ± 0.01	0.38 ± 0.01	0.14 ± 0.02	0.25 ± 0.03
77	44.33	1658	1658	BSO	Valerianol	0.29 ± 0.03	0.39 ± 0.08	0.16 ± 0.02	0.15 ± 0.01	0.35 ± 0.04	0.37 ± 0.02	0.55 ± 0.05
78	44.51	1662	1663	BSO	*7-epi-α* Eudesmol	0.08 ± 0.01	0.14 ± 0.02	0.14 ± 0.01	0.15 ± 0.01	0.08 ± 0.02	0.08 ± 0.01	0.08 ± 0.01

Abbreviations: AM—aliphatic monoterpene; MM—monocyclic monoterpene; BM—bi- and tricyclic monoterpene; AMO—aliphatic monoterpenoid; MMO—monocyclic monoterpenoid; BMO—bi- and tricyclic monoterpenoid; AS—aliphatic sesquiterpene; MS—monocyclic sesquiterpene; BS—bi- and tricyclic sesquiterpene; ASO—aliphatic sesquiterpenoid; MSO—monocyclic sesquiterpenoid; BSO—bi- and tricyclic sesquiterpenoid; OT—other. SD—standard deviation; Exp. RI—experimental retention index; Ref. RI—literature data; t_R_—retention time.

### 3.2. Multivariate Analysis

**Figure 2:**
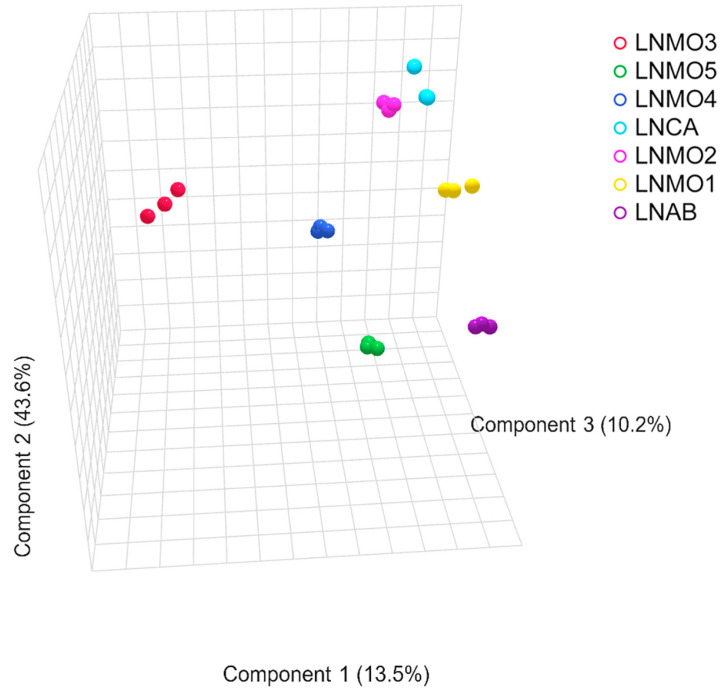
A 3D score plot of the PLS-DA of the essential oils of *L. nobilis*.

**Figure 3:**
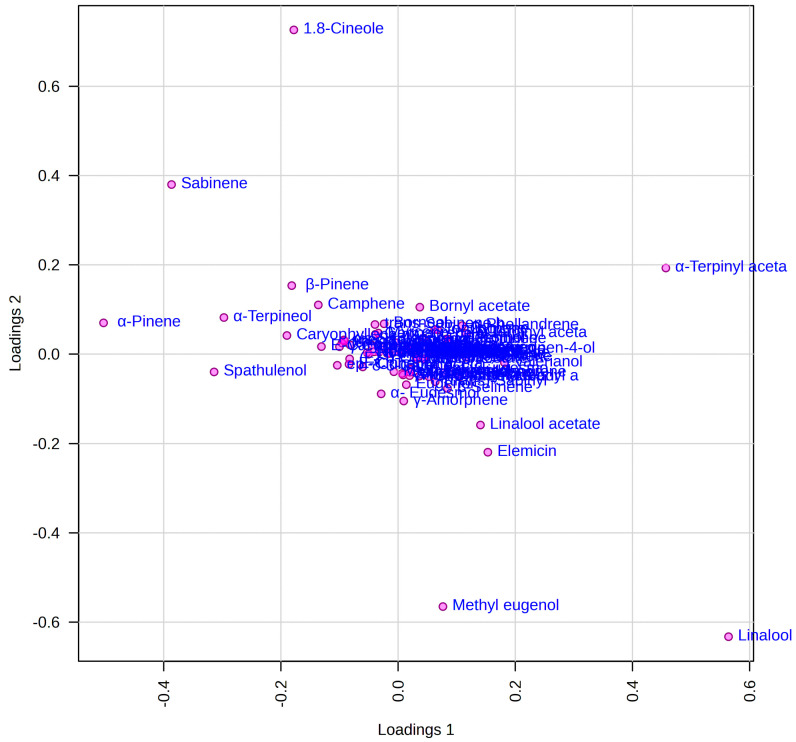
Loading plot of PLS-DA.

Multivariate analyses of the untargeted and targeted data were performed to establish metabolite differences in the analyzed EOs based on the GC data. Principal Component Analysis (PCA) was primarily used to categorize data and to find correlations between samples and variables. The PLS-DA was calculated to assess the similarities/differences between the essential oils of *L. nobilis* L. and to highlight the metabolites that contribute the most to the variety of differentiation by means of Variable Importance in Projection analysis (VIP). The first three components accounted for 67.3% (PC1 = 13.5%, PC2 = 43.6%, and PC3 = 10.2%), and the model was determined by R2 and Q2 values. The score plot of the PLS-DA (Figure 2 and Figure 3) shows seven different clusters, with a slight similarity between LNMO2 and LNCA.

In order to investigate the metabolites that differentiate the EOs the most and are responsible for cluster separation, the VIP analysis was performed, showing the top ten metabolites with a VIP value > 1 along PC1 (Figure S2) and along PC2 (Figure S3). In the loading plot of the PLS-DA where the variables are displayed (Figure 3), α-pinene, sabinene, 1,8-cineole, α-terpinyl acetate, linalool, and methyl eugenol represent the main metabolites that contribute to the discrimination of samples.

In fact, the major discriminating metabolites observed in the loading plot are in accordance with the VIP plots, which explain the differentiation of the varieties shown in the score plot of the PLS-DA.

### 3.3. Total Phenols, Antioxidant Potential, and Reducing Activity

It is known that phenolic compounds are one of the most important classes of natural antioxidants, and their richness affects the antioxidant activity of plant tissues [92]. In this study, we aimed to assess whether EOs’ in vitro antioxidant activity was also attributable to non-phenolic components together with the influence of altitude, as previously reported [93,94]. Several in vitro tests are often used to assess the antioxidant capacity of EOs based on various chemical methodologies. Hence, the combination of different complementary assays can give a clearer idea of the antioxidant activity [95]. Two different assays were used in this study, which showed differences in antioxidant activity between the various EO accessions. The potential antioxidant capacity of seven samples was measured using 1,1-diphenyl-2-picrylhydrazyl (DPPH) [96] and ferric-reducing antioxidant power (FRAP) [77] model systems.

The IC_50_ values of the antioxidant activity of the EOs from the leaves of *L. nobilis* and ascorbic acid as a positive control are displayed in Table 2. In the DPPH assay, the reduction of the stable radical DPPH to the yellow-colored DPPH-H is employed to measure the capability of an antioxidant molecule to act as a donor of hydrogen atoms or electrons. In this radical scavenging assay, all EO samples displayed an IC_50_ ranging from 411.55 mg/mL (LNMO2) to 535.90 mg/mL (LNMO4), with a weak activity when compared with the value of standard ascorbic acid (3.75 μg/mL). In the FRAP assay, the values found for EOs (from 0.51 to 0.27 mmol TE/g) showed a low reducing activity with respect to the reference ascorbic acid (11.10 mmol TE/g). Generally, the antioxidant capacity is positively correlated with total phenol content (TPC) levels; so, in EOs, the higher TPC, as in LNMO1 (11.36 mg GAE/g), corresponds to the best reducing capacity (0.51mmol TE/g FRAP) with good antioxidant activity (IC_50_ 465.58 μg/mL) in the DPPH assay. The sample LNMO4 has the lowest TPC (6.60 mg GAE/g), which corresponds to a weak reducing power (0.27 mmol TE/g) in the FRAP assay. *L. nobilis* grown in plain areas and near the sea (LNMO1) presents the highest TPC, while there is a high degree of similarity between samples collected in areas of increasing altitude. Interestingly, LNCA (about 35 m a.s.l.) has the lowest TPC, and the harvest site is far from the sea in an inland hilly area. Since EOs are complex mixtures often composed of many different molecules, their biological activity is difficult to rationalize; therefore, the numerous papers reported on the antioxidant activity of EOs usually assume synergism, antagonism, and additivity between the various compounds [97]. 

**Table 2:** Total phenolic content (TPC) and antioxidant capacity of EOs of *L. nobilis* assessed by DPPH and FRAP methods. IC_50_: the concentration of the extract that inhibits 50% of the radical activity. TE: Trolox equivalent. GAE: gallic acid equivalent. Each value is a mean ± SD of triplicate analysis.

Sample	TPC (mg GAE/g)	DPPH IC_50_ (μg/mL)	FRAP (mmol TE/g)
**LNMO1**	11.36 ± 0.23	465.58 ± 2.80	0.51 ± 0.01
**LNMO2**	10.55 ± 0.24	411.55 ± 13.11	0.32 ± 0.01
**LNMO3**	10.13 ± 0.14	423.04 ± 26.24	0.30 ± 0.01
**LNMO4**	6.60 ± 0.29	535.90 ± 28.02	0.27 ± 0.01
**LNMO5**	10.24 ± 0.30	505.91 ± 10.57	0.40 ± 0.01
**LNCA**	7.97 ± 0.24	519.90 ± 17.12	0.27 ± 0.01
**LNAB**	8.28 ± 0.17	423.76 ± 10.86	0.30 ± 0.01
**Ascorbic acid**	-	3.75 ± 0.01	11.10 ± 0.2

Abbreviations: LNMO1: sample collected in Petacciato (CB); LNMO2: sample collected in Campobasso (CB); LNMO3: sample collected in Isernia (IS), LNMO4: sample collected in Carpinone (IS); LNMO5: sample collected in Capracotta (IS) LNAB: sample collected in Rosciano (Pescara); LNCA: sample collected in Santa Maria Capua Vetere (Caserta).

We assume that the main cause of the moderate antioxidant activity of our EOs is a modest content of phenolic compounds represented only by a few phenylpropanoids such as eugenol and methyl eugenol, two benzene derivatives where the -OH group of eugenol is replaced by the -OCH_3_ group in methyl eugenol. In the EO samples, the amount of methyl eugenol is rather high in the LNMO1, LNMO4, and LNABA samples (10.54, 14.5, and 10.86, respectively), while the content of eugenol ranges from 1.97 (LNMO4) to 4.12 (LNMO1).

Recently, Nenadis et al. [98] demonstrated, by means of theoretical and experimental tests, that methyl eugenol exhibits a low scavenging potential compared to that of eugenol against the DPPH radical. We also observed that cultivation in the entire Molise region did not produce a significant difference in EO yield between the lowland and mountainous–hilly areas, ranging from 0.24% (LNMO3) to 0.5% in LNMO4. The highest yield was found for the EO sample LNAB (1.1%) collected in the neighboring region.

In conclusion, our results show a high degree of similarity among the EOs for TPC, scavenging potential, and reducing activity. Only LNMO1 collected at sea level differs from the other samples, followed by LNMO2, LNMO3, and LNMO5 collected between 400 and 1400 m in the Molise region, while LNCA and LNAB from the neighboring regions are very similar and have a lower TPC content.

### 3.4. Cell-Free sEH Activity Assay

Numerous studies indicate that sEH inhibitors could offer significant therapeutic benefits in treating and managing inflammatory diseases and associated pain [99,100]. Structure–activity relationship studies suggest that lipophilicity plays a critical role in determining the inhibition of the sEH enzyme, as observed in synthetic 1,3-disubstituted ureas, carbamates, and amides [101]. In fact, a typical sEH hydrolase site inhibitor should have a “urea/amide-like portion” able to interact with the catalytic triad (i.e., Tyr383, Tyr466, and Asp335) and a “hydrophobic portion” able to interact with a large L-shaped hydrophobic region (i.e., Phe267, Phe387, Leu408, Met419, and Leu428) at the entrance of the catalytic binding site (vide infra) [102].

Recently, several natural products have already been identified as sEH inhibitors, including natural ureas, triterpenoids, flavonoids, and phenylpropionic acids [103]. Natural products can be modified by introducing polar groups to improve solubility, offering new avenues for developing sEH inhibitors for clinical applications as an alternative chemical scaffold to synthetic compounds. In some cases, extracts from plant origins were tested, and after verifying their ability to inhibit the sEH protein, each component was isolated to determine which one was responsible for the activity on the target [104,105].

**Figure 4:**
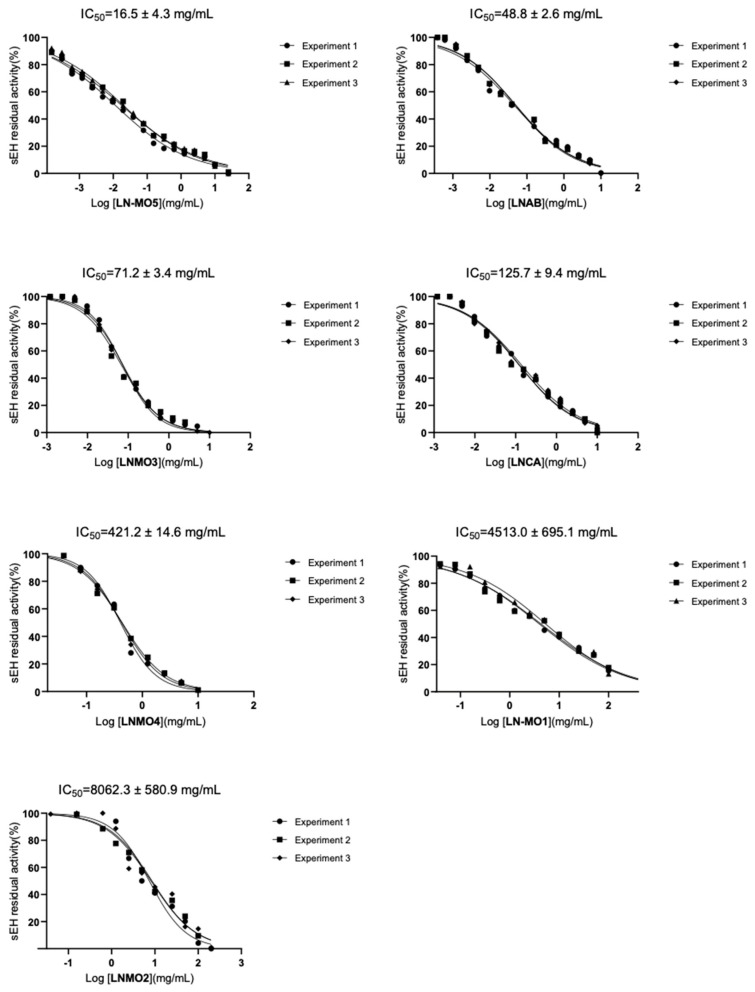
Concentration−response curves for analysis of seven *L. nobilis* L. essential oils on sEH isolated enzyme. Data are expressed as percentage of control (100%) and means with SD; n = 3.

Thus, in line with previous studies on natural extracts, we evaluated the potential ability of the EOs of seven Italian *Laurus nobilis* L. varieties to inhibit the sEH enzyme. The different samples of *Laurus nobilis* L. essential oils were solubilized in EtOH, and their inhibitory potencies were measured against human sEH using a fluorescent assay, as described by Colarusso et al. [106] (see Materials and Methods Section). The half-maximal inhibitory concentration (IC_50_) values were determined for each oil starting at a concentration of 10 mg/mL, and adjustments were made to higher or lower concentrations depending on the initial results. All of the oils were able to inhibit the sEH enzyme (Table 3 and Figure 4), with LNMO5, LNAB, and LNMO3 possessing the most potent inhibitory activities (IC_50_ = 16.5 ± 4.3, 48.8 ± 2.6, and 71.2 ± 3.4 mg/mL, respectively). LNCA and LNMO4 showed moderate activity in inhibiting sEH (IC_50_ = 125.7 ± 9.4 and 421.2 ± 14.6 mg/mL, respectively). Instead, sEH activity inhibition was less effective for the oils LNMO1 and LNMO2 (IC_50_ > 500 mg/mL).

Interestingly, these results point out the promising anti-inflammatory activity of the EOs, considering that they are a mixture of multiple volatile phytochemicals, each of which does not exert its own unique chemical profile. The observed biological activities, in fact, are due to the co-presence of compounds that could act in a synergetic or antagonistic manner toward the target. Moreover, these results are compatible with a fragment virtual screening approach of natural compounds because we speculate that the observed inhibitory sEH hydrolase activity could be ascribed to the most abundant volatile secondary metabolites (vide supra). The latter could be used as a starting point for further studies on compounds isolated from EOs, exploring further pathways of inflammation-related lipid mediators.

### 3.5. Computational Studies to Analyze Interactions between sEH and the Main Chemical Constituents of the EOs

**Figure 5:**
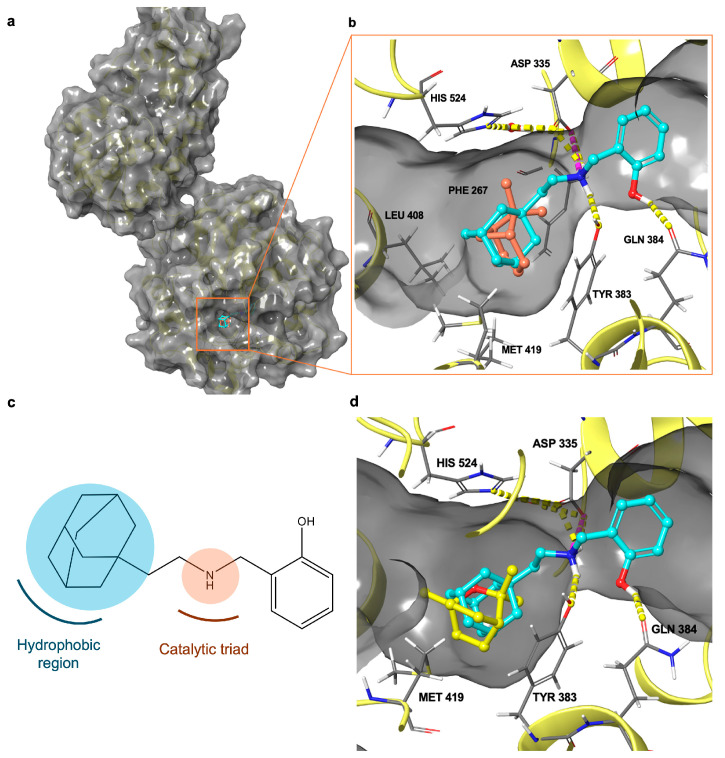
(**a**) Three-dimensional structure of sEH in complex with known inhibitor 4A0 and α-pinene. (**b**) Superposition of binding modes of α-pinene (colored by atom type: C orange) and known inhibitor 4A0 (colored by atom type: C cyan, O red, N blue, polar H light gray). Hydrogen bonds are shown as dotted yellow lines. (**c**) Schematic representation of pharmacophoric portion of known inhibitor 4A0. (**d**) Superposition of known inhibitor 4A0 (colored by atom type: C cyan, O red, N blue, polar H light gray) with 1,8-cineole (colored by atom type: C yellow, O red, polar H light gray).

To rationalize the inhibitory activity of EOs on sEH, we performed computational studies on the most abundant volatile secondary metabolites (≥ 1.0%, Table 1) identified by GC-FID and GC/MS analysis. Molecular docking experiments allowed for the analysis of the interactions at the molecular level between the enzyme and this small set of natural products (Figure 1 and Figures S4–S7, related to the area (%) as ≥1.0% and ≥0.5%, respectively) found in greater concentrations in each sample of EOs. As mentioned above, a well-known sEH hydrolase site inhibitor (Figure 5c) should have a chemical group (“urea/amide-like portion”) able to establish hydrogen bonds with the catalytic triad (i.e., Tyr383, Tyr466, and Asp335) and a “hydrophobic portion” able to interact with a large L-shaped hydrophobic region (i.e., Phe267, Phe387, Leu408, Met419, and Leu428) at the entrance of the catalytic binding site (vide supra). These chemical features are, in fact, respected by the known inhibitor 4A0 used as a reference during our computational studies. More specifically, we analyzed the in silico data, considering these EO phytochemicals as fragment hits derived through virtual screening of natural compounds. Notably, we have hypothesized that these natural fragment hits could interact, in a similar manner with the known inhibitor 4A0, with an L-shaped hydrophobic tunnel, hampering access to the endogenous substrate of the enzyme hydrolase site (Figure 5 and Table S2). In fact, these compounds exhibit fragment-like properties with a shape similar to that of the adamantyl group of the known inhibitor 4A0 [78]. Thus, in this inhibitor, these molecules consist of a sterically encumbered hydrophobic group that can occupy the lipophilic region of the enzyme. For the sake of simplicity, we used the binding mode of α-pinene and 1,8-cineole (Figure 5), two of the compounds present in comparable concentrations in the EOs analyzed, to corroborate our hypothesis. In more detail, it fits into the opening of the enzyme binding site by occupying the hydrophobic region surrounded by Leu408, Met419, and Phe267, in the same way as the adamantyl group of the known inhibitor 4A0 co-crystallized with sEH (PDB ID: 4Y2X [78]).

**Figure 6:**
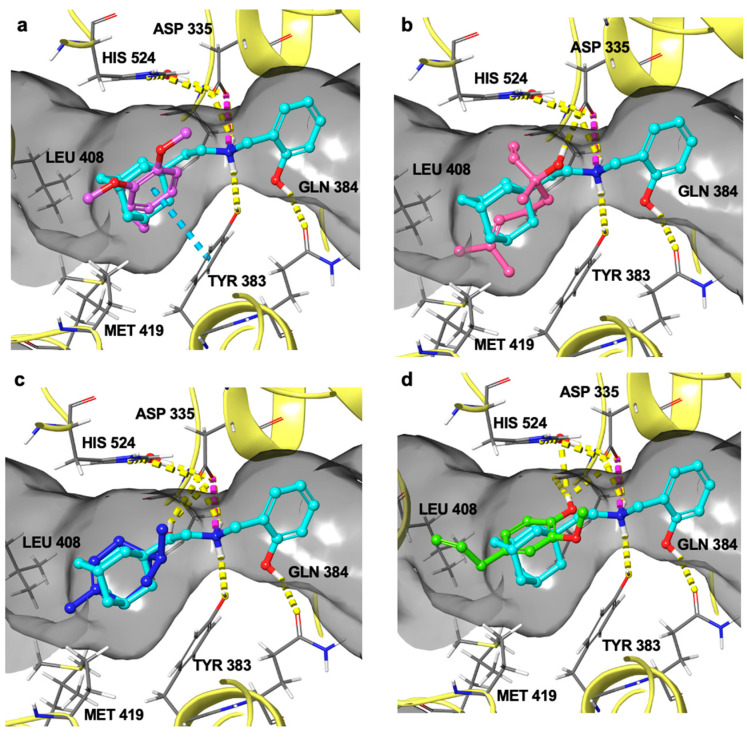
Superposition of known inhibitor 4A0 (colored by atom type: C cyan, O red, N blue, polar H light gray) with (**a**) methyl eugenol (colored by atom type: C blue, O red, polar H light gray); (**b**) linalool (colored by atom type: C pink, O red, polar H light gray); (**c**) terpinene-4-ol (colored by atom type: C blue, O red, polar H light gray); (**d**) eugenol (colored by atom type: C green, O red, polar H light gray). Hydrogen bonds are shown as dotted yellow lines, π-π stacking interactions are shown as dotted blue lines, and salt bridges are shown as dotted purple lines.

Interestingly, methyl eugenol, linalool, terpinen-4-ol, and eugenol are the compounds that, in addition to occupying the hydrophobic L-shaped pocket, establish further interactions with amino acids of the catalytic site of the enzyme (Figure 6). Thus, these data are in agreement with the more significant observed inhibitory activity against sEH, where they have been found in higher concentrations in the most active essential oils, namely LNMO5, LNAB, and LNMO1.

In detail, methyl eugenol, abundant in LNMO5 and present in slightly lower concentrations in LNAB and LNMO1, binds to the active site of sEH by occupying the hydrophobic pocket and establishing an interaction with Try383, one of the amino acids in the catalytic triad of the enzyme (Figure 6a). Linalool, detected in higher concentrations in LNMO5 and LNAB, forms an H-bond with Asp335, belonging to the catalytic triad (Figure 6b).

Also, terpinen-4-ol, a component mainly contained in the LNMO5, LNAB, and LNMO1 samples, fits into the hydrophobic region and, through its -OH group, establishes an H-bond with Asp335 (Figure 6c). Finally, eugenol, found in higher concentrations in LNMO5 and LNMO1, forms H-bonds with Asp335 and with His524, which coordinates a water molecule critical in the reaction mechanism catalyzed by the enzyme (Figure 6d).

Based on these data, it is possible to assume that the more potent inhibitory activity observed in the LNMO5, LNAB, and LNMO1 samples could be attributed to the synergistic effect of the compounds that exhibit a better binding mode by occupying the hydrophobic L-shaped pocket and partially interact with the catalytic triad of sEH.

## 4. Conclusions

The chemical composition of seven EOs of *L. nobilis* was analyzed, revealing that 1,8-cineole is the predominant component in all samples, followed by methyl-eugenol, α-terpinyl acetate, linalool, and sabinene. Using multivariate data analysis, it was possible to differentiate EOs based on the composition of their metabolites. The total phenolic content (TPC) was determined, and then, the antioxidant potency of the samples was evaluated using the DPPH and FRAP methods. LNMO1 showed the best reducing capacity correlated with the highest total phenol content (TPC); LNMO4 showed the lowest TPC and weakest reducing power. The EOs showed moderate antioxidant activity, probably due to the low content of phenolic compounds represented only by eugenol, methyl eugenol, and elemicin.

Considering these data, which clarify the chemical composition of hydrophobic metabolites in all samples, we tested the EOs as inhibitors of the soluble epoxide hydrolase enzyme (sEH) to verify their anti-inflammatory properties. All essential oils demonstrated the ability to inhibit the sEH enzyme in concentrations ranging from 16.5 ± 4.3 to 8062.3 ± 580.9 mg/mL. These results demonstrate the promising anti-inflammatory activity of EOs, which is attributable to the synergistic or antagonistic activity that co-present compounds exert on the target. Furthermore, computational investigations suggest that the most abundant volatile secondary metabolites (≥1.0%) represent natural fragment hits derived from these EOs, potentially capable of occupying the L-shaped hydrophobic region of the protein in a manner similar to the adamantyl group of the known inhibitor. Interestingly, among these, methyl eugenol, linalool, eugenol, and terpinen-4-ol have emerged as the most promising natural fragments because they establish interactions with amino acids belonging to both the hydrophobic pocket and the catalytic triad of the enzyme, and this could be responsible for the different trend in inhibitory activity of the seven laurel EOs.

These findings are crucial for the development of naturally derived chemical scaffolds as alternatives to synthetic enzyme inhibitors. In our study, however, it is important to consider that the EOs are mixtures of different components with probably lower activity than pure single compounds. Therefore, our future goal will be to extract the most significant compounds of the EOs of *L. nobilis* L., test them singularly on the sEH enzyme, and then create, from these fragment hits, semi-synthetic derivatives that can further improve their biological effects.

## Figures and Tables

**Table 3:** IC_50_ (Half-maximal inhibitory concentration) values of *Laurus nobilis* essential oils against sEH isolated enzyme. Values are shown as means of three separate experiments ± SD. AUDA was used as reference compound.

Tested Oils	IC_50_ ± SD (mg/mL)
LNMO5	16.5 ± 4.3
LNAB	48.8 ± 2.6
LNMO3	71.2 ± 3.4
LNCA	125.7 ± 9.4
LNMO4	421.2 ± 14.6
LNMO1	4513.0 ± 695.1
LNMO2	8062.3 ± 580.9
AUDA (known inhibitor)	0.068 ± 0.003

Abbreviations: LNMO1: sample collected in Petacciato (CB); LNMO2: sample collected in Campobasso (CB); LNMO3: sample collected in Isernia (IS), LNMO4: sample collected in Carpinone (IS); LNMO5: sample collected in Capracotta (IS) LNAB: sample collected in Rosciano (Pescara); LNCA: sample collected in Santa Maria Capua Vetere (Caserta); IC_50_: (Half-maximal inhibitory concentration).

## Data Availability

The original contributions presented in the study are included in the article; further inquiries can be directed to the corresponding author.

## Supplementary Materials

The following supporting information can be downloaded at: https://www.mdpi.com/article/10.3390/foods13142282/s1, Table S1. List of terpenes in *L. nobilis* essential oils (area%) and yield%; Figure S1. A 2D score plot of the PLS-DA of the essential oils of *L. nobilis* varieties; Figure S2. VIP plot in component 1; Figure S3. VIP plot in component 2; Figure S4. The binding mode of 4A0 (in cyan) superimposed to its pose (in green) in the crystal structure of sEH (PDB code: 4Y2X); Table S2. Interactions of the known inhibitor 4A0 and major chemical components with the enzyme sEH. Figure S5. Binding mode of (a) a-terpinyl acetate (9.16–13%), (b) elemicin (2.05–0.57%), (c) a-terpineol (2.91–0.91%), (d) spathulenol (1.76–0.57%), (e) sabinene (10.57–4.85%), (f) myrcene (1.02–0.38%), and (g) b-pinene (3.77–2.44%); Figure S6. Binding mode of (a) γ-amorphene (0.73–0.08%), (b) valerianol (0.55–0.15%), (c) E-caryophyllene (0.79–0.32%), (d) caryophyllene oxide (0.83–0.16%), (e) d-cadinene (0.55–0.11%), (f) camphene (0.90–0.10%), (g) bornyl acetate (0.82–0.13%), and (h) b-elemene (0.79–0.20%). Figure S7. Binding mode of (a) d-3-carene (0.58–0.10%), (b) methyl chavicol (0.93–0.07%), (c) a-phellandrene (0.79–0.03%), (d) g-terpinene (0.83–0.68%), (e) E-cinnamyl acetate (0.96–0.03%), (f) linalool acetate (0.57–0.06%), (g) o-cymene (0.63%), and (h) d-terpinyl acetate (0.76–0.40%); Figure S8. IC_50_ of AUDA (reference compound) on sEH. Data are expressed as means of three experiments ± SD.

## References

[1] Reuter S., Gupta S.C., Chaturvedi M.M., Aggarwal B.B. (2010). Oxidative stress, inflammation, and cancer: How are they linked?. Free Radic. Biol. Med..

[2] Vona R., Pallotta L., Cappelletti M., Severi C., Matarrese P. (2021). The impact of oxidative stress in human pathology: Focus on gastrointestinal disorders. Antioxidants.

[3] Chatterjee S., Dziubla T., Butterfield D.A. (2016). Chapter Two—Oxidative Stress, Inflammation, and Disease. Oxidative Stress and Biomaterials.

[4] Pahwa R., Goyal A., Jialal I. (2023). Chronic Inflammation.

[5] Furman D., Campisi J., Verdin E., Carrera-Bastos P., Targ S., Franceschi C., Ferrucci L., Gilroy D.W., Fasano A., Miller G.W. (2019). Chronic inflammation in the etiology of disease across the life span. Nat. Med..

[6] Chen L., Deng H., Cui H., Fang J., Zuo Z., Deng J., Li Y., Wang X., Zhao L. (2018). Inflammatory responses and inflammation-associated diseases in organs. Oncotarget.

[7] Wang Y.X.J., Ulu A., Zhang L.N., Hammock B. (2010). Soluble epoxide hydrolase in atherosclerosis. Curr. Atheroscler. Rep..

[8] Newman D.J., Cragg G.M. (2020). Natural Products as Sources of New Drugs over the Nearly Four Decades from 01/1981 to 09/2019. J. Nat. Prod..

[9] He J., Wang C., Zhu Y., Ai D. (2016). Soluble epoxide hydrolase: A potential target for metabolic diseases. J. Diabetes.

[10] Das Mahapatra A., Choubey R., Datta B. (2020). Small molecule soluble epoxide hydrolase inhibitors in multitarget and combination therapies for inflammation and cancer. Molecules.

[11] Sun C.-P., Zhang X.-Y., Morisseau C., Hwang S.H., Zhang Z.-J., Hammock B.D., Ma X.-C. (2021). Discovery of Soluble Epoxide Hydrolase Inhibitors from Chemical Synthesis and Natural Products. J. Med. Chem..

[12] Gazzillo E., Terracciano S., Ruggiero D., Potenza M., Chini M.G., Lauro G., Fischer K., Hofstetter R.K., Giordano A., Werz O. (2022). Repositioning of Quinazolinedione-Based Compounds on Soluble Epoxide Hydrolase (sEH) through 3D Structure-Based Pharmacophore Model-Driven Investigation. Molecules.

[13] Seca A.M.L., Moujir L. (2020). Natural compounds: A dynamic field of applications. Appl. Sci..

[14] Boshtam M., Asgary S., Kouhpayeh S., Shariati L., Khanahmad H. (2017). Aptamers Against Pro- and Anti-Inflammatory Cytokines: A Review. Inflammation.

[15] Mucha P., Skoczyńska A., Małecka M., Hikisz P., Budzisz E. (2021). Overview of the antioxidant and anti-inflammatory activities of selected plant compounds and their metal ions complexes. Molecules.

[16] Miguel M.G. (2010). Antioxidant and anti-inflammatory activities of essential oils: A short review. Molecules.

[17] Giacometti J., Bursać Kovačević D., Putnik P., Gabrić D., Bilušić T., Krešić G., Stulić V., Barba F.J., Chemat F., Barbosa-Cánovas G. (2018). Extraction of bioactive compounds and essential oils from mediterranean herbs by conventional and green innovative techniques: A review. Food Res. Int..

[18] Awada F., Hamade K., Kassir M., Hammoud Z., Mesnard F., Rammal H., Fliniaux O. (2023). *Laurus nobilis* Leaves and Fruits: A Review of Metabolite Composition and Interest in Human Health. Appl. Sci..

[19] Jaradat N., Abualhasan M., Hawash M., Qadi M., Al-Maharik N., Abdallah S., Mousa A., Zarour A., Arar M., Sobuh S. (2023). Chromatography analysis, in light of vitro antioxidant, antidiabetic, antiobesity, anti-inflammatory, antimicrobial, anticancer, and three-dimensional cancer spheroids’ formation blocking activities of *Laurus nobilis* aromatic oil from Palestine. Chem. Bio Technol. Agric..

[20] Dobroslavić E., Garofulić I.E., Zorić Z., Pedisić S., Dragović-Uzelac V. (2021). Polyphenolic characterization and antioxidant capacity of *Laurus nobilis* L. Leaf extracts obtained by green and conventional extraction techniques. Processes.

[21] Anzano A., de Falco B., Grauso L., Motti R., Lanzotti V. (2022). Laurel, *Laurus nobilis* L.: A review of its botany, traditional uses, phytochemistry and pharmacology. Phytochem. Rev..

[22] Paparella A., Nawade B., Shaltiel-Harpaz L., Ibdah M. (2022). A Review of the Botany, Volatile Composition, Biochemical and Molecular Aspects, and Traditional Uses of *Laurus nobilis* L. Plants.

[23] Bianchi A. (2015). The Mediterranean aromatic plants and their culinary use. Nat. Prod. Res..

[24] Dinsmore S., Grams M.-K., Couris R.R. (2018). Bay Leaf: Leaf of the European Laurel: An Overview of Potential Benefits and Safety. Nutr. Today.

[25] Ordoudi S.A., Papapostolou M., Nenadis N., Mantzouridou F.T., Tsimidou M.Z. (2022). Bay Laurel (*Laurus nobilis* L.) Essential Oil as a Food Preservative Source: Chemistry, Quality Control, Activity Assessment, and Applications to Olive Industry Products. Foods.

[26] Batool S., Khera R.A., Hanif M.A., Ayub M.A., Hanif M.A., Nawaz H., Khan M.M., Byrne H.J. (2019). Bay Leaf. Medicinal Plants of South Asia: Novel Sources for Drug Discovery.

[27] Caputo L., Nazzaro F., Souza L.F., Aliberti L., De Martino L., Fratianni F., Coppola R., De Feo V. (2017). *Laurus nobilis*: Composition of essential oil and its biological activities. Molecules.

[28] Alejo-Armijo A., Altarejos J., Salido S. (2017). Phytochemicals and Biological Activities of Laurel Tree (*Laurus nobilis*). Nat. Prod. Commun..

[29] Mansour O., Darwish M., Ismail G., Douba Z.A.-A., Ismaeel A., Eldair K. (2018). Review Study on the Physiological Properties and Chemical Composition of the *Laurus nobilis*. Pharm. Chem. J..

[30] Fidan H., Stefanova G., Kostova I., Stankov S., Damyanova S., Stoyanova A., Zheljazkov V.D. (2019). Chemical Composition and Antimicrobial Activity of *Laurus nobilis* L. Essential oils from Bulgaria. Molecules.

[31] Georgiev E., Stoyanova A. (2006). A Guide for the Specialist in the Aromatic Industry.

[32] Sayyah M., Saroukhani G., Peirovi A., Kamalinejad M. (2003). Analgesic and anti-inflammatory activity of the leaf essential oil of *Laurus nobilis* Linn. Phytother. Res..

[33] Sayyah M., Valizadeh J., Kamalinejad M. (2002). Anticonvulsant activity of the leaf essential oil of *Laurus nobilis* against pentylenetetrazole- and maximal electroshock-induced seizures. Phytomedicine.

[34] De Marino S., Borbone N., Zollo F., Ianaro A., Di Meglio P., Iorizzi M. (2004). Megastigmane and Phenolic Components from *Laurus nobilis* L. Leaves and Their Inhibitory Effects on Nitric Oxide Production. J. Agric. Food Chem..

[35] Muñiz-Márquez D.B., Rodríguez R., Balagurusamy N., Carrillo M.L., Belmares R., Contreras J.C., Nevárez G.V., Aguilar C.N. (2014). Phenolic content and antioxidant capacity of extracts of *Laurus nobilis* L., Coriandrum sativum L. and Amaranthus hybridus L. CYTA J. Food.

[36] Dias M.I., Barros L., Dueñas M., Alves R.C., Oliveira M.B.P.P., Santos-Buelga C., Ferreira I.C.F.R. (2014). Nutritional and antioxidant contributions of *Laurus nobilis* L. leaves: Would be more suitable a wild or a cultivated sample?. Food Chem..

[37] Vinha A.F., Guido L.F., Costa A.S.G., Alves R.C., Oliveira M.B.P.P. (2015). Monomeric and oligomeric flavan-3-ols and antioxidant activity of leaves from different *Laurus* sp. Food Funct..

[38] Senchenko S.P., Nasukhova N.M., Agova L.A., Konovalov D.A. (2016). Use of Micellar Electrokinetic Chromatography to Analyze Sesquiterpene Lactones from *Laurus nobilis* L. Pharm. Chem. J..

[39] Barla A., Topçu G., Öksüz S., Tümen G., Kingston D.G.I. (2007). Identification of cytotoxic sesquiterpenes from *Laurus nobilis* L. Food Chem..

[40] Matsuda H., Kagerura T., Toguchida I., Ueda H., Morikawa T., Yoshikawa M. (1999). Inhibitory effects of sesquiterpenes from Bat Leaf on nitric oxide production in lipopolysaccharide-activated macrophages: Structure requirement and role of heat shock protein induction. Life Sci..

[41] Dobroslavić E., Repajić M., Dragović-Uzelac V., Garofulić I.E. (2022). Isolation of *Laurus nobilis* Leaf Polyphenols: A Review on Current Techniques and Future Perspectives. Foods.

[42] Li Q., Wang Z., Xie Y., Hu H. (2020). Antitumor activity and mechanism of costunolide and dehydrocostus lactone: Two natural sesquiterpene lactones from the Asteraceae family. Biomed. Pharmacother..

[43] Butturini E., Carcereri De Prati A., Boriero D., Mariotto S. (2019). Natural Sesquiterpene Lactones Enhance Chemosensitivity of Tumor Cells through Redox Regulation of STAT3 Signaling. Oxid. Med. Cell Longev..

[44] Pacifico S., Gallicchio M., Lorenz P., Potenza N., Galasso S., Marciano S., Fiorentino A., Stintzing F.C., Monaco P. (2013). Apolar *Laurus nobilis* leaf extracts induce cytotoxicity and apoptosis towards three nervous system cell lines. Food Chem. Toxicol..

[45] Saab A.M., Tundis R., Loizzo M.R., Lampronti I., Borgatti M., Gambari R., Menichini F., Esseily F., Menichini F. (2012). Antioxidant and antiproliferative activity of *Laurus nobilis* L. (Lauraceae) leaves and seeds essential oils against K562 human chronic myelogenous leukaemia cells. Nat. Prod. Res..

[46] Dall’acqua S., Viola G., Giorgetti M., Loi M.C., Innocenti G. (2006). Two New Sesquiterpene Lactones from the Leaves of *Laurus nobilis*; *Chem*. Pharm. Bull..

[47] Panza E., Tersigni M., Iorizzi M., Zollo F., De Marino S., Festa C., Napolitano M., Castello G., Ialenti A., Ianaro A. (2011). Lauroside B, a Megastigmane Glycoside from *Laurus nobilis* (Bay Laurel) Leaves, Induces Apoptosis in Human Melanoma Cell Lines by Inhibiting NF-κB Activation. J. Nat. Prod..

[48] Autiero I., Roviello G.N. (2023). Interaction of Laurusides 1 and 2 with the 3C-like Protease (M^pro^) from Wild-Type and Omicron Variant of SARS-CoV-2: A Molecular Dynamics Study. Int. J. Mol. Sci..

[49] Chahal K.K., Singh D.K., Panchbhaiya A., Singh N., Kaur M., Bhardwaj U., Singla N., Kaur A. (2017). A review on chemistry and biological activities of *Laurus nobilis* L. essential oil. J. Pharmacogn. Phytochem..

[50] Siriken B., Yavuz C., Guler A. (2018). Antibacterial Activity of *Laurus nobilis*: A review of literature. Med. Sci. Discov..

[51] Mssillou I., Agour A., El Ghouizi A., Hamamouch N., Lyoussi B., Derwich E. (2020). Chemical Composition, Antioxidant Activity, and Antifungal Effects of Essential Oil from *Laurus nobilis* L. Flowers Growing in Morocco. J. Food Qual..

[52] Dearlove R.P., Greenspan P., Hartle D.K., Swanson R.B., Hargrove J.L. (2008). Inhibition of Protein Glycation by Extracts of Culinary Herbs and Spices. J. Med. Food.

[53] Basak S.S., Candan F. (2013). Effect of *Laurus nobilis* L. Essential Oil and its Main Components on α-glucosidase and Reactive Oxygen Species Scavenging Activity. Iran. J. Pharm. Res..

[54] Al-Kalaldeh J.Z., Abu-Dahab R., Afifi F.U. (2010). Volatile oil composition and antiproliferative activity of *Laurus nobilis*, *Origanum syriacum*, *Origanum vulgare*, and *Salvia triloba* against human breast adenocarcinoma cells. Nutr. Res..

[55] Ercin E., Kecel-Gunduz S., Gok B., Aydin T., Budama-Kilinc Y., Kartal M. (2022). *Laurus nobilis* L. Essential Oil-Loaded PLGA as a Nanoformulation Candidate for Cancer Treatment. Molecules.

[56] Pilipović K., Jurišić Grubešić R., Dolenec P., Kučić N., Juretić L., Mršić-Pelčić J. (2023). Plant-Based Antioxidants for Prevention and Treatment of Neurodegenerative Diseases: Phytotherapeutic Potential of *Laurus nobilis*, *Aronia melanocarpa*, and Celastrol. Antioxidants.

[57] Ferreira A., Proença C., Serralheiro M.L.M., Araújo M.E.M. (2006). The in vitro screening for acetylcholinesterase inhibition and antioxidant activity of medicinal plants from Portugal. J. Ethnopharmacol..

[58] Cherrat L., Espina L., Bakkali M., García-Gonzalo D., Pagán R., Laglaoui A. (2014). Chemical composition and antioxidant properties of *Laurus nobilis* L. and *Myrtus communis* L. essential oils from Morocco and evaluation of their antimicrobial activity acting alone or in combined processes for food preservation. J. Sci. Food Agric..

[59] Amorati R., Foti M.C., Valgimigli L. (2013). Antioxidant Activity of Essential Oils. J. Agric. Food Chem..

[60] Alimi D., Hajri A., Jallouli S., Sebai H. (2021). In vitro acaricidal activity of essential oil and crude extracts of *Laurus nobilis*, (Lauraceae) grown in Tunisia, against arthropod ectoparasites of livestock and poultry: *Hyalomma scupense* and *Dermanyssus gallinae*. Vet. Parasitol..

[61] Furtado R., Baptista J., Lima E., Paiva L., Barroso J.G., Rosa J.S., Oliveira L. (2014). Chemical composition and biological activities of *Laurus* essential oils from different Macaronesian Islands. Biochem. Syst. Ecol..

[62] Adişen E., Önder M. (2007). Allergic contact dermatitis from *Laurus nobilis* L oil induced by massage. Contact Dermatitis.

[63] Kıvrak Ş., Göktürk T., Kıvrak İ. (2017). Assessment of Volatile Oil Composition, Phenolics and Antioxidant Activity of Bay (*Laurus nobilis* L) Leaf and Usage in Cosmetic Applications. Inter. J. Sec Metabolite.

[64] Diedrich C., da Silva L.D., Sari R., de Cristo Borges G.C., Muniz H.S., de Lima V.A., Oldoni T.L.C., Carpes S.T. (2021). Bioactive compounds extraction of *Croton lechleri* barks from Amazon forest using chemometrics tools. J. King Saud. Univ. Sci..

[65] Council of Europe (2004). European Pharmacopoeia.

[66] El S.N., Karagozlu N., Karakaya S., Sahın S. (2014). Antioxidant and Antimicrobial Activities of Essential Oils Extracted from *Laurus nobilis* L. Leaves by Using Solvent-Free Microwave and Hydrodistillation. Food Nutr. Sci..

[67] Sparkman O.D. (2005). Identification of essential oil components by gas chromatography/quadrupole mass spectroscopy. J. Am. Soc. Mass. Spectrom..

[68] Van Den Dool H., Kratz P.D. (1963). A generalization of the retention index system including linear temperature programmed gas—Liquid partition chromatography. J. Chromatogr. A.

[69] Kováts E. (1965). Gas chromatographic characterization of organic substances in the retention index system. Advan Chromatogr..

[70] McLafferty F.W. (2000). Wiley Registry of Mass Spectral Data, with NIST Spectral Data CD Rom.

[71] NIST, EPA, NIH (2005). Mass Spectral Library.

[72] Grob R.L., Kaiser M.A., Grob R.L., Barry E.F. (2004). Qualitative and Quantitative Analysis by Gas Chromatography. Modern Practice of Gas Chromatography.

[73] Ruiz-Perez D., Guan H., Madhivanan P., Mathee K., Narasimhan G. (2020). So you think you can PLS-DA?. BMC Bioinformatics.

[74] Want E., Masson P., Metz T.O. (2011). Processing and Analysis of GC/LC-MS-Based Metabolomics Data BT—Metabolic Profiling: Methods and Protocols. Metabolic Profiling.

[75] Michiu D., Socaciu M.I., Fogarasi M., Jimborean A.M., Ranga F., Mureşan V., Semeniuc C.A. (2022). Implementation of an Analytical Method for Spectrophotometric Evaluation of Total Phenolic Content in Essential Oils. Molecules.

[76] Wei A., Shibamoto T. (2010). Antioxidant/Lipoxygenase Inhibitory Activities and Chemical Compositions of Selected Essential Oils. J. Agric. Food Chem..

[77] Benzie I.F.F., Strain J.J. (1996). The Ferric Reducing Ability of Plasma (FRAP) as a Measure of “Antioxidant Power”: The FRAP Assay. Anal. Biochem..

[78] Amano Y., Tanabe E., Yamaguchi T. (2015). Identification of N-ethylmethylamine as a novel scaffold for inhibitors of soluble epoxide hydrolase by crystallographic fragment screening. Bioorg Med. Chem..

[79] Schrödinger Release 2021-1: Protein Preparation Wizard; Epik, Schrödinger, LLC, New York, NY, 2021; Impact, Schrödinger, LLC, New York, NY; Prime, Schrödinger, LLC, New York, NY, 2021. https://www.schrodinger.com/citations/.

[80] Madhavi Sastry G., Adzhigirey M., Day T., Annabhimoju R., Sherman W. (2013). Protein and ligand preparation: Parameters, protocols, and influence on virtual screening enrichments. J. Comput. Aided Mol. Des..

[81] Schrödinger, LLC (2021). LigPrep.

[82] Friesner R.A., Murphy R.B., Repasky M.P., Frye L.L., Greenwood J.R., Halgren T.A., Sanschagrin P.C., Mainz D.T. (2006). Extra Precision Glide: Docking and Scoring Incorporating a Model of Hydrophobic Enclosure for Protein−Ligand Complexes. J. Med. Chem..

[83] Yang Y., Yao K., Repasky M.P., Leswing K., Abel R., Shoichet B.K., Jerome S. (2021). V Efficient Exploration of Chemical Space with Docking and Deep Learning. J. Chem. Theory Comput..

[84] Halgren T.A., Murphy R.B., Friesner R.A., Beard H.S., Frye L.L., Pollard W.T., Banks J.L. (2004). Glide: A New Approach for Rapid, Accurate Docking and Scoring. 2. Enrichment Factors in Database Screening. J. Med. Chem..

[85] Friesner R.A., Banks J.L., Murphy R.B., Halgren T.A., Klicic J.J., Mainz D.T., Repasky M.P., Knoll E.H., Shelley M., Perry J.K. (2004). Glide: A New Approach for Rapid, Accurate Docking and Scoring. 1. Method and Assessment of Docking Accuracy. J. Med. Chem..

[86] Schrödinger, LLC (2021). Glide.

[87] Amin G., Sourmaghi M.S., Jaafari S., Hadjagaee R., Yazdinezhad A. (2007). Influence of Phenological Stages and Method of Distillation on Iranian Cultivated Bay Leaves Volatile Oil. Pak. J. Biol. Sci..

[88] Rodilla J.M., Tinoco M.T., Morais J.C., Gimenez C., Cabrera R., Martín-Benito D., Castillo L., Gonzalez-Coloma A. (2008). *Laurus novocanariensis* essential oil: Seasonal variation and valorization. Biochem. Syst. Ecol..

[89] Burt S. (2004). Essential oils: Their antibacterial properties and potential applications in foods—A review. Int. J. Food Microbiol..

[90] Ovidi E., Masci V.L., Zambelli M., Tiezzi A., Vitalini S., Garzoli S. (2021). *Laurus nobilis*, *Salvia sclarea* and *Salvia officinalis* essential oils and hydrolates: Evaluation of liquid and vapor phase chemical composition and biological activities. Plants.

[91] Nabila B., Piras A., Fouzia B., Falconieri D., Kheira G., Fedoul F.F., Majda S.R. (2022). Chemical composition and antibacterial activity of the essential oil of *Laurus nobilis* leaves. Nat. Prod. Res..

[92] Fantasma F., Samukha V., Saviano G., Chini M.G., Iorizzi M., Caprari C. (2024). Nutraceutical Aspects of Selected Wild Edible Plants of the Italian Central Apennines. Nutraceuticals.

[93] Yılmaz B., Deniz İ. (2017). The Effects of Cultivation Area and Altitude Variation on the Composition of Essential Oil of *Laurus nobilis* L. Grown in Eastern, Western and Central Karadeniz Region. Int. J. Second. Metab..

[94] Chen X., Shang S., Yan F., Jiang H., Zhao G., Tian S., Chen R., Chen D., Dang Y. (2023). Antioxidant Activities of Essential Oils and Their Major Components in Scavenging Free Radicals, Inhibiting Lipid Oxidation and Reducing Cellular Oxidative Stress. Molecules.

[95] Kotha R.R., Tareq F.S., Yildiz E., Luthria D.L. (2022). Oxidative Stress and Antioxidants—A Critical Review on In Vitro Antioxidant Assays. Antioxidants.

[96] Heimler D., Vignolini P., Dini M.G., Romani A. (2005). Rapid Tests to Assess the Antioxidant Activity of *Phaseolus vulgaris* L. Dry Beans. J. Agric. Food Chem..

[97] Hsouna A.B., Halima N.B., Abdelkafi S., Hamdi N. (2013). Essential Oil from *Artemisia phaeolepis*: Chemical Composition and Antimicrobial Activities. J. Oleo Sci..

[98] Nenadis N., Papapostolou M., Tsimidou M.Z. (2021). Suggestions on the contribution of methyl eugenol and eugenol to bay laurel (*Laurus nobilis* L.) essential oil preservative activity through radical scavenging. Molecules.

[99] Schmelzer K.R., Kubala L., Newman J.W., Kim I.-H., Eiserich J.P., Hammock B.D. (2005). Soluble epoxide hydrolase is a therapeutic target for acute inflammation. Proc. Natl. Acad. Sci. USA.

[100] Wagner K.M., McReynolds C.B., Schmidt W.K., Hammock B.D. (2017). Soluble epoxide hydrolase as a therapeutic target for pain, inflammatory and neurodegenerative diseases. Pharmacol. Ther..

[101] Morisseau C., Goodrow M.H., Newman J.W., Wheelock C.E., Dowdy D.L., Hammock B.D. (2002). Structural refinement of inhibitors of urea-based soluble epoxide hydrolases. Biochem. Pharmacol..

[102] Gomez G.A., Morisseau C., Hammock B.D., Christianson D.W. (2004). Structure of Human Epoxide Hydrolase Reveals Mechanistic Inferences on Bifunctional Catalysis in Epoxide and Phosphate Ester Hydrolysis. Biochemistry.

[103] Lee G.H., Oh S.J., Lee S.Y., Lee J.Y., Ma J.Y., Kim Y.H., Kim S.K. (2014). Discovery of soluble epoxide hydrolase inhibitors from natural products. Food Chem. Toxicol..

[104] Cuong T., Anh H., Thu Hường T., Khanh P., Ha V., Tran M.H., Kim Y.H., Cuong N. (2019). Identification of Soluble Epoxide Hydrolase Inhibitors from the Seeds of *Passiflora edulis* Cultivated in Vietnam. Nat. Prod. Sci..

[105] Kim J.H., Morgan A., Tai B., Van D., Cuong N., Kim Y.H. (2015). Inhibition of soluble epoxide hydrolase activity by compounds isolated from the aerial parts of *Glycosmis stenocarpa*. J. Enzyme Inhib. Med. Chem..

[106] Colarusso E., Potenza M., Lauro G., Chini M.G., Sepe V., Zampella A., Fischer K., Hofstetter R.K., Werz O., Bifulco G. (2022). Thiazolidin-4-one-based compounds interfere with the eicosanoid biosynthesis pathways by mPGES-1/sEH/5-LO multi-target inhibition. Eur. J. Med. Chem. Rep..

