# Supplementary material for: Essential Oils of Laurus nobilis L.: From Chemical Analysis to In Silico Investigation of Anti-Inflammatory Activity by Soluble Epoxide Hydrolase (sEH) Inhibition
Source: Foods. 2024 Jul 20;13(14):2282. doi: 10.3390/foods13142282 (PMC11276180; doi:10.3390/foods13142282)
Supplement: Supplementary file 1 [file foods-13-02282-s001.zip › foods-3097680-supplementary.pdf]

Supplementary Materials

Essential Oils of *Laurus nobilis* L.: From Chemical Analysis to In Silico Investigation of Anti-Inflammatory Activity by Soluble Epoxide Hydrolase (sEH) Inhibition

Francesca Fantasma [1], Vadym Samukha [1], Michela Aliberti [2], Ester Colarusso [2], Maria Giovanna Chini [1 *], Gabriella Saviano [1], Vincenzo De Felice [1], Gianluigi Lauro [2], Agostino Casapullo [2], Giuseppe Bifulco [2 ,*,†] and Maria Iorizzi [1,†].

[1] Department of Biosciences and Territory, University of Molise, Contrada Fonte Lappone, 86090 Pesche (IS), Italy; fantasma@unimol.it (F.F.); v.samukha@studenti.unimol.it (V.S.); saviano@unimol.it (G.S.); defelice@unimol.it (V.D.F.); iorizzi@unimol.it (M.I.).

[2] Department of Pharmacy, University of Salerno, Via Giovanni Paolo II 132, 84084 Fisciano (SA), Italy; mialiberti@unisa.it (M.A.); ecolarusso@unisa.it (E.C.); glauro@unisa.it (G.L.); casapullo@unisa.it (A.C.).

*Correspondence: mariagiovanna.chini@unimol.it (M.G.C.); bifulco@unisa.it (G.B.);

† These authors contributed equally to this work.

Tel.: +39-0874404132 (M.G.C.);+39-089969741 (G.B.).

Table of contents

Table S1. List of terpenes in *L. nobilis* essential oils (area %) and yield % S2

Figure S1. A 2D score plot of the PLS-DA of the essential oils of *L. nobilis* varieties. S3

Figure S2. VIP plot in component 1. ....S4

Figure S3. VIP plot in component 2. ....S4

Re-docking of the known inhibitor 4A0 co-crystallized in the protein structure of sEH..S5

Figure S4. The binding mode of 4A0 (in cyan) superimposed to its pose (in green) in the crystal structure of sEH (PDB code: 4Y2X) S5

Table S2. Interactions of the known inhibitor 4A0 and major chemical components with the enzyme sEH. S5

Figure S5. Binding mode of a) α-terpinyl acetate (9.16 – 13 %), b) elemicin (2.05 - 0.57 %), c) α-terpineol (2.91 – 0.91 %) , d) spathulenol (1.76 – 0.57 %), e) sabinene (10.57 – 4.85 %), f) myrcene (1.02 – 0.38 %), and g) β-pinene (3.77 – 2.44 %). Hydrogen bonds are shown as dotted yellow lines. S6

Figure S6. Binding mode of a) γ-amorphene (0.73 – 0.08 %), b) valerianol (0.55 – 0.15 %), c) E-caryophyllene (0.79 – 0.32 %), d) caryophyllene oxide (0.83 – 0.16 %), e) δ-cadinene (0.55 – 0.11 %) , f) camphene (0.90 – 0.10 %), g) bornyl acetate (0.82 – 0.13 %), and h) β-elemene (0.79 – 0.20 %)...S6

Figure S7. Binding mode of a) δ-3-carene (0.58 – 0.10 %), b) methyl chavicol (0.93 – 0.07 %), c) α-phellandrene (0.79 – 0.03 %), d) γ-terpinene (0.83 – 0.68 %), e) E-cinnamyl acetate (0.96 – 0.03 %), f) linalool acetate (0.57 – 0.06 %), g) o-cymene (0.63 %), and h) δ-terpinyl acetate (0.76 – 0.40 %). π-π stacking interactions are shown as dotted blue lines. S7

Figure S8. IC_{50} of AUDA (reference compound) on sEH. Data are expressed as means of three experiments ± SD. S7

References S7

Table S1. List of terpenes in *L. nobilis* essential oils (area%) and yield%

	Class	LNMO1	LNMO2	LNMO3	LNMO4	LNMO5	LNCA	LNAB
Aliphatic monoterpenes	AM	0.52	1.05	0.94	1.34	0.74	1.16	0.85
Monocyclic monoterpenes	MM	3.03	1.47	1.81	1.42	1.41	1.93	1.73
Bi – and tricyclic monoterpenes	BM	16.78	19.52	23.66	15.79	13.14	19.1	15.27
**Monoterpenes**	**M**	**20.33**	**22.04**	**26.41**	**18.55**	**15.29**	**22.19**	**17.85**
Aliphatic monoterpenoids	AMO	1.45	4.35	1.37	3.19	12.74	3.93	11.1
Monocyclic monoterpenoids	MMO	16.29	15.24	13.32	14.19	12.98	17.73	13.72
Bi – and tricyclic monoterpenoids	BMO	42.43	45.38	40.75	41.43	32.38	43.06	34.42
**Monoterpenoids**	**MO**	**60.17**	**64.79**	**55.44**	**58.81**	**58.1**	**64.72**	**59.25**
Aliphatic sesquiterpenes	AS	-	-	-	-	-	-	-
Monocyclic sesquiterpenes	MS	0.51	0.53	0.9	0.61	0.48	1.18	1.32
Bi – and tricyclic sesquiterpenes	BS	1.17	0.9	2.15	1.5	1.62	1.89	3.3
**Sesquiterpenes**	**S**	**1.68**	**1.43**	**3.05**	**2.11**	**2.1**	**3.07**	**4.62**
Aliphatic sesquiterpenoids	ASO	-	-	-	-	-	-	-
Monocyclic sesquiterpenoids	MSO	0.10	-	-	-	0.06	-	-
Bi – and tricyclic sesquiterpenoids	BSO	1.91	2.2	3.39	0.83	2.38	1.29	2.11
**Sesquiterpenoids**	**SO**	**2.01**	**2.2**	**3.39**	**0.83**	**2.44**	**1.29**	**2.11**
**Others**	**OT**	**16.11**	**8.65**	**10.58**	**18.8**	**21.57**	**7.74**	**15.35**
**EO's yield**		**0.46%**	**0.4%**	**0.24%**	**0.5%**	**0.46%**	**0.37%**	**1.1%**

Abbreviations: AM- aliphatic monoterpenes; MM- monocyclic monoterpenes; BM-bi-and tricyclic monoterpenes; AMO- aliphatic monoterpenoids; MMO- monocyclic monoterpenoids; BMO- bi-and tricyclic monoterpenoids; AS- aliphatic sesquiterpenes; MS- monocyclic sesquiterpenes; BS- bi- and tricyclic sesquiterpenes; ASO- aliphatic sesquiterpenoids; MSO- monocyclic sesquiterpenoids; BSO- bi- and tricyclic sesquiterpenoids, OT- others.

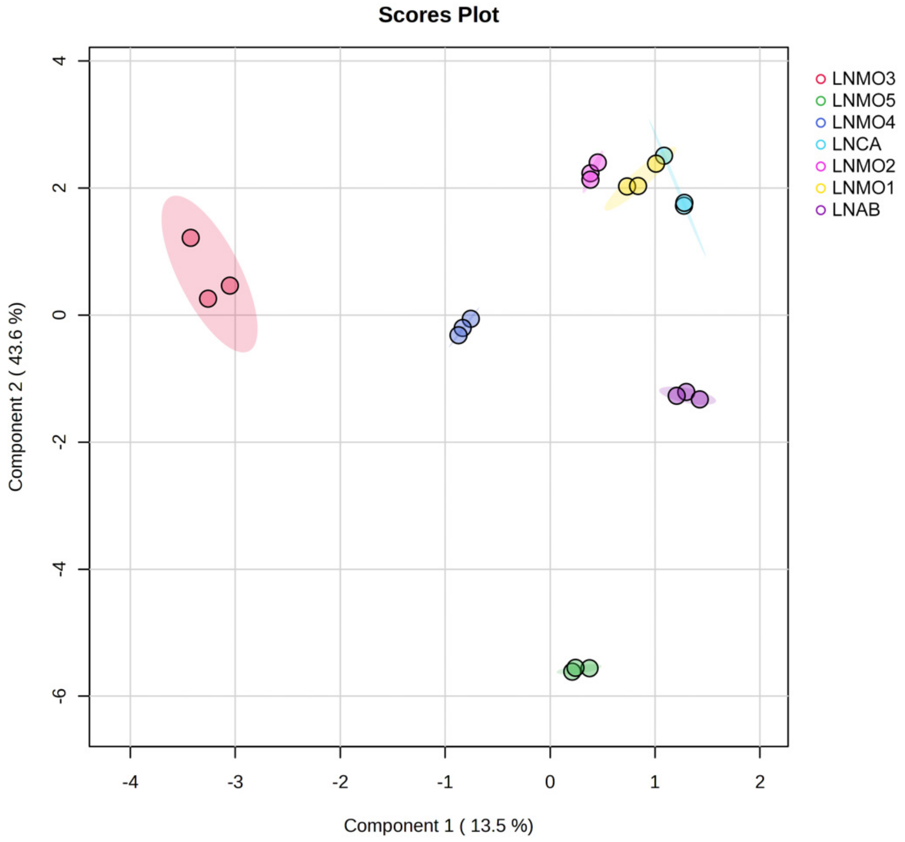

Figure S1. A 2D score plot of the PLS-DA of the essential oils of *L. nobilis* varieties.

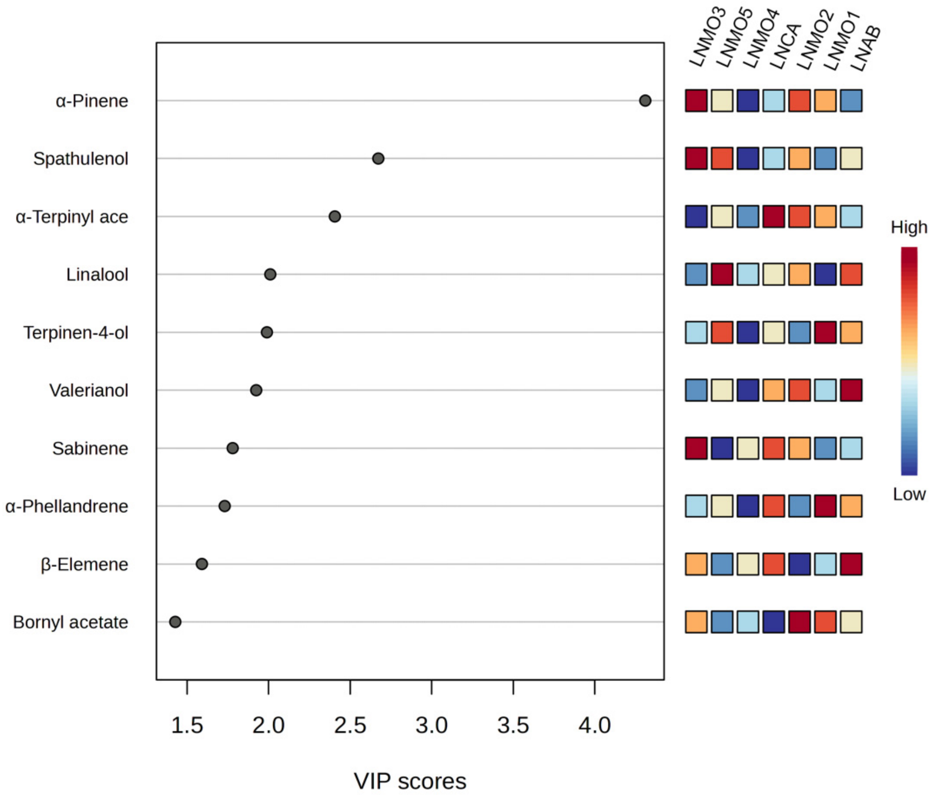

Figure S2. VIP plot in component 1.

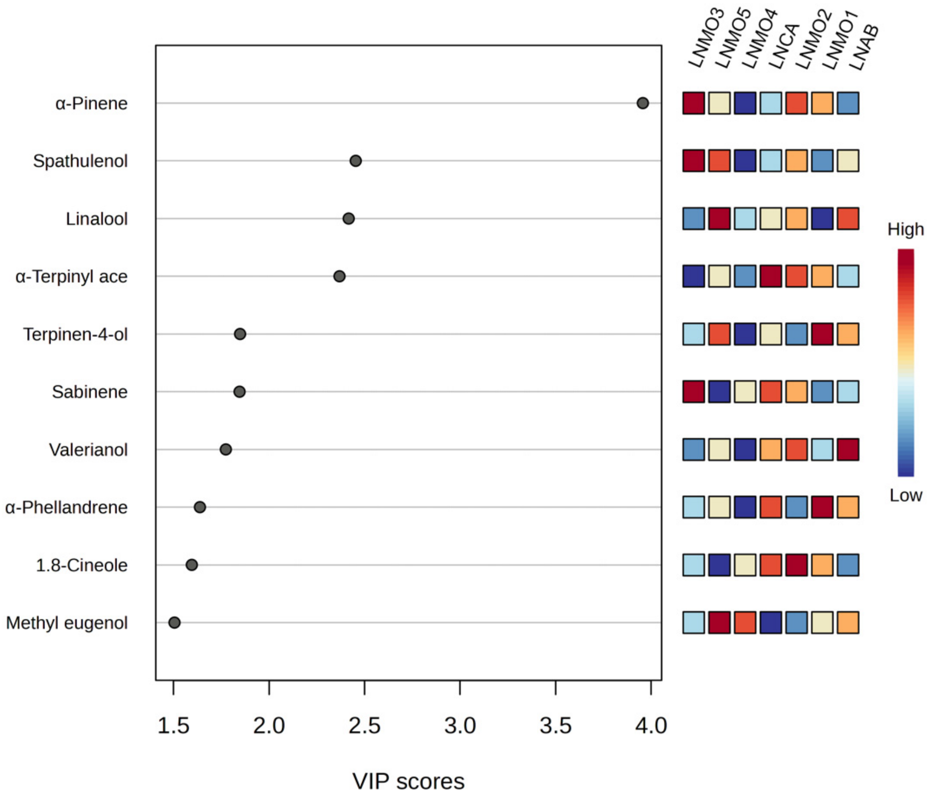

Figure S3. VIP plot in component 2.

Re-docking of the known inhibitor 4A0 co-crystallized in the protein structure of sEH

The compound 4A0 co-crystallized in the protein structure of sEH (PDB code: 4Y2X[1]) as reference to define molecular docking parameters. Specifically, Glide software (Schrödinger Suite) [2–6] was used in the Extra Precision (XP) mode to reproduce the binding mode of the compound ADUA as reported in the crystal structure. The generated molecular docking pose replicated a similar binding mode observed for the co-crystallized form of this ligand, as shown in Figure S4.

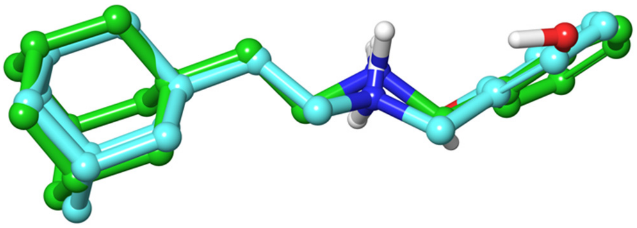

Figure S4. The binding mode of 4A0 (in cyan) superimposed to its pose (in green) in the crystal structure of sEH (PDB code: 4Y2X).

Table S2. Interactions of the known inhibitor 4A0 and major chemical components with the enzyme sEH.

Compounds	Type of interactions	Amino acids
**4A0**	Hydrogen bonds	Tyr383, His524, Gln 384
	Salt bridges	Asp 335
	Hydrophobic contacts	Leu408, Met419, Phe267
**1,8-Cineole**	Hydrophobic contacts	Leu408, Met419, Phe267
**α-Terpinyl acetate**	Hydrogen bonds	His524
	Hydrophobic contacts	Leu408, Met419, Phe267
**Sabinene**	Hydrophobic contacts	Leu408, Met419, Phe267
**Eugenol**	Hydrogen bonds	Asp335, His524
	Hydrophobic contacts	Leu408, Met419, Phe267
**α-Pinene**	Hydrophobic contacts	Leu408, Met419, Phe267
**Linalool**	Hydrogen bonds	Asp 335
	Hydrophobic contacts	Leu408, Met419, Phe267
**Methyl eugenol**	π-π interactions	Tyr 383
	Hydrophobic contacts	Leu408, Met419, Phe267
**Terpinen-4-ol**	Hydrogen bonds	Asp 335
	Hydrophobic contacts	Leu408, Met419, Phe267

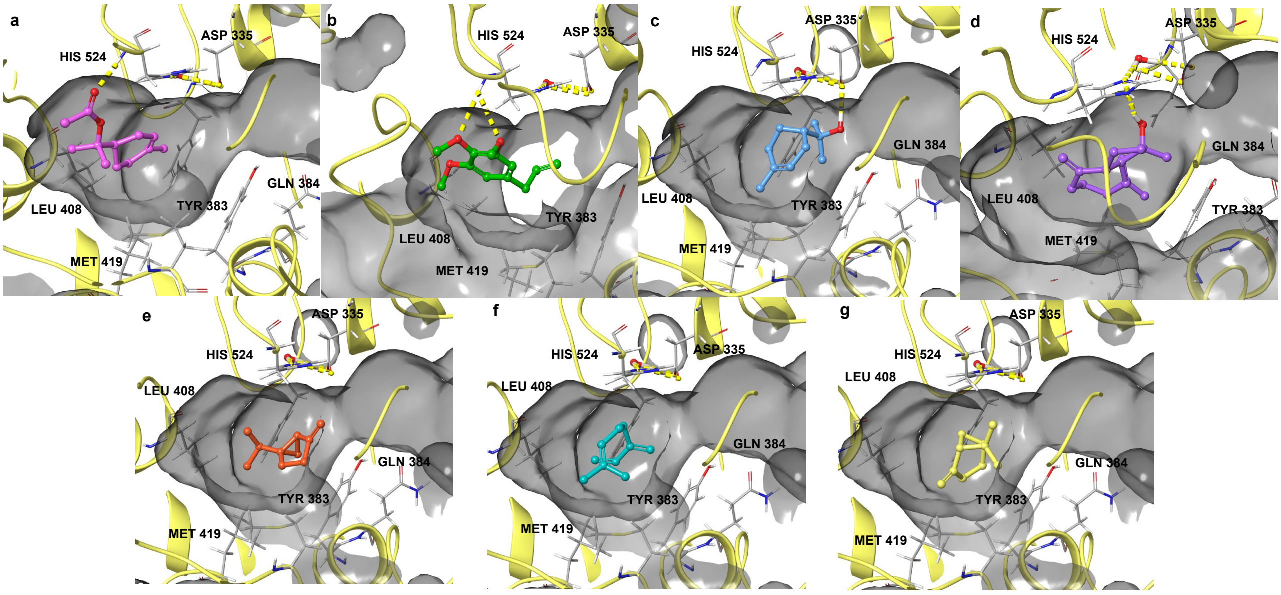

Figure S5. Binding mode of (a) α-terpinyl acetate (9.16–13%), (b) elemicin (2.05 − 0.57%), (c) α-terpineol (2.91 − 0.91%), (d) spathulenol (1.76 − 0.57%), (e) sabinene (10.57 − 4.85%), (f) myrcene (1.02 − 0.38%), and (g) β-pinene (3.77 − 2.44%). Hydrogen bonds are shown as dotted yellow lines.

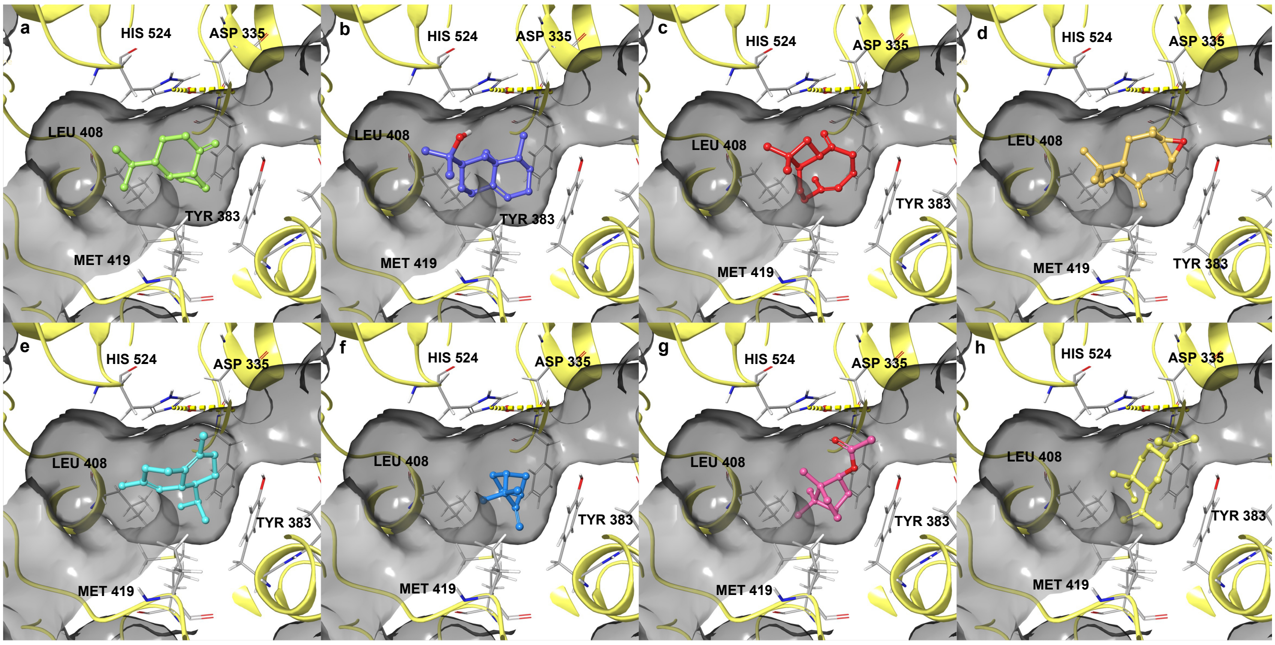

Figure S6. Binding mode of (a) γ-amorphene (0.73 − 0.08%), (b) valerianol (0.55 − 0.15%), (c) E-caryophyllene (0.79 − 0.32%), (d) caryophyllene oxide (0.83 − 0.16%), (e) δ-cadinene (0.55 − 0.11%), (f) camphene (0.90 − 0.10%), (g) bornyl acetate (0.82 − 0.13%), and (h) β-elemene (0.79 − 0.20%).

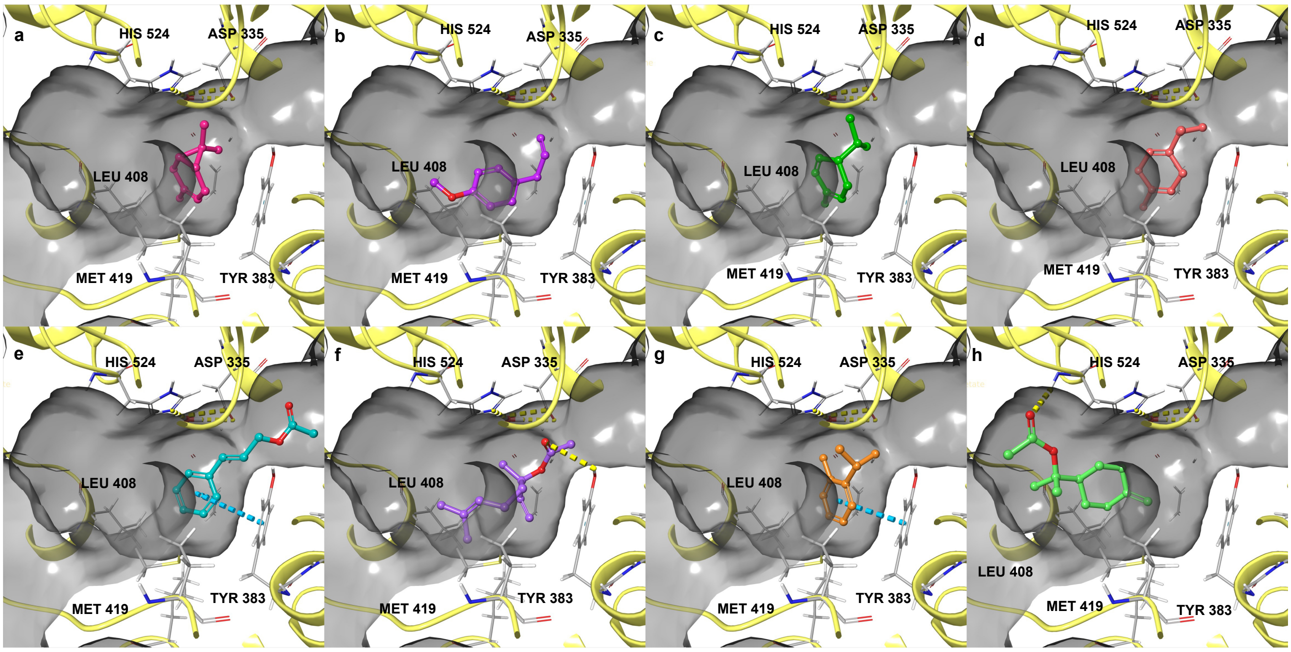

Figure S7. Binding mode of (a) δ-3-carene (0.58 − 0.10%), (b) methyl chavicol (0.93 − 0.07%), (c) α-phellandrene (0.79 − 0.03%), (d) γ-terpinene (0.83 − 0.68%), (e) E-cinnamyl acetate (0.96 − 0.03%), (f) linalool acetate (0.57 − 0.06%), (g) o-cymene (0.63%), and (h) δ-terpinyl acetate (0.76 − 0.40%). π-π stacking interactions are shown as dotted blue lines.

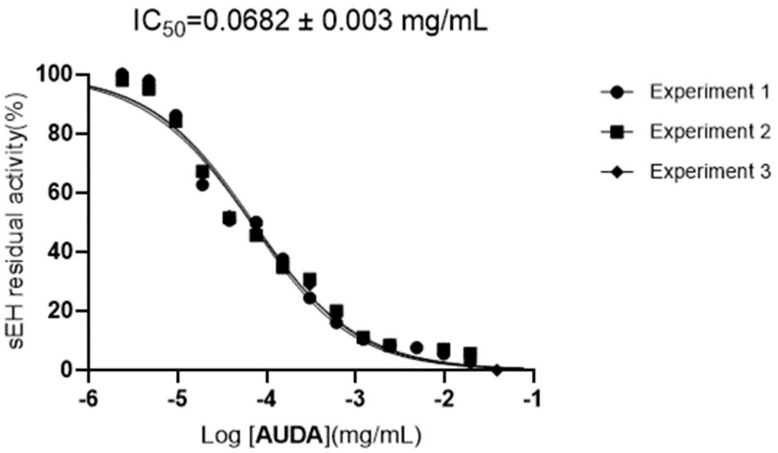

Figure S8. IC_{50} of AUDA (reference compound) on sEH. Data are expressed as means of three experiments ± SD.

References

1. Amano, Y.; Tanabe, E.; Yamaguchi, T. Identification of N-ethylmethylamine as a novel scaffold for inhibitors of soluble epoxide hydrolase by crystallographic fragment screening. *Bioorg Med Chem* **2015**, *23*, 2310–2317.

2. Friesner, R. A.; Murphy, R. B.; Repasky, M. P.; Frye, L. L.; Greenwood, J. R.; Halgren, T. A.; Sanschagrin, P. C.; Mainz, D. T. Extra Precision Glide: Docking and Scoring Incorporating a Model of Hydrophobic Enclosure for Protein−Ligand Complexes. *J Med Chem* **2006**, *49*, 6177–6196.

3. Yang, Y.; Yao, K.; Repasky, M. P.; Leswing, K.; Abel, R.; Shoichet, B. K.; Jerome, S. V Efficient Exploration of Chemical Space with Docking and Deep Learning. *J Chem Theory Comput* **2021**, *17*, 7106–7119.

4. Schrödinger Release 2021-1: Glide, Schrödinger, LLC, New York, NY, 2021.

5. Halgren, T. A.; Murphy, R. B.; Friesner, R. A.; Beard, H. S.; Frye, L. L.; Pollard, W. T.; Banks, J. L. Glide: A New Approach for Rapid, Accurate Docking and Scoring. 2. Enrichment Factors in Database Screening. *J Med Chem* **2004**, *47*, 1750–1759.

6. Friesner, R. A.; Banks, J. L.; Murphy, R. B.; Halgren, T. A.; Klicic, J. J.; Mainz, D. T.; Repasky, M. P.; Knoll, E. H.; Shelley, M.; Perry, J. K.; et al. Glide: A New Approach for Rapid, Accurate Docking and Scoring. 1. Method and Assessment of Docking Accuracy. *J Med Chem* **2004**, *47*, 1739–1749.
